# Molecular Ecology and Natural History of Simian Foamy Virus Infection in Wild-Living Chimpanzees

**DOI:** 10.1371/journal.ppat.1000097

**Published:** 2008-07-04

**Authors:** Weimin Liu, Michael Worobey, Yingying Li, Brandon F. Keele, Frederic Bibollet-Ruche, Yuanyuan Guo, Paul A. Goepfert, Mario L. Santiago, Jean-Bosco N. Ndjango, Cecile Neel, Stephen L. Clifford, Crickette Sanz, Shadrack Kamenya, Michael L. Wilson, Anne E. Pusey, Nicole Gross-Camp, Christophe Boesch, Vince Smith, Koichiro Zamma, Michael A. Huffman, John C. Mitani, David P. Watts, Martine Peeters, George M. Shaw, William M. Switzer, Paul M. Sharp, Beatrice H. Hahn

**Affiliations:** 1 Departments of Medicine and Microbiology, University of Alabama at Birmingham, Birmingham, Alabama, United States of America; 2 University of Arizona, Tucson, Arizona, United States of America; 3 Gladstone Institute for Virology and Immunology, University of California at San Francisco, San Francisco, California, United States of America; 4 Faculties of Sciences, University of Kisangani, Democratic Republic of Congo; 5 Institut de Recherche pour le Développement (IRD) and University of Montpellier 1, Montpellier, France; 6 Projet Prevention du Sida ou Cameroun (PRESICA), Yaoundé, Cameroun; 7 Centre International de Recherches Medicales de Franceville (CIRMF), Franceville, Gabon; 8 Max-Planck Institute for Evolutionary Anthropology, Leipzig, Germany; 9 Gombe Stream Research Centre, The Jane Goodall Institute, Tanzania; 10 Department of Anthropology, University of Minnesota, Minneapolis, Minnesota, United States of America; 11 Jane Goodall Institute's Center for Primate Studies, Department of Ecology, Evolution and Behavior, University of Minnesota, St. Paul, Minnesota, United States of America; 12 Antioch New England Graduate School, Keene, New Hampshire, United States of America; 13 The Gorilla Organization, Kigali, Rwanda; 14 Great Ape Research Institute, Hayashibara Biochemical Laboratories, Okayama, Japan; 15 Section of Ecology, Primate Research Institute, Kyoto University, Aichi, Japan; 16 Department of Anthropology, University of Michigan, Ann Arbor, Michigan, United States of America; 17 Department of Anthropology, Yale University, New Haven, Connecticut, United States of America; 18 Laboratory Branch, National Center for HIV/AIDS, STD, and TB Prevention, Centers for Disease Control and Prevention, Atlanta, Georgia, United States of America; 19 Institute of Evolutionary Biology, University of Edinburgh, Edinburgh, United Kingdom; The Pennsylvania State University, United States of America

## Abstract

Identifying microbial pathogens with zoonotic potential in wild-living primates can be important to human health, as evidenced by human immunodeficiency viruses types 1 and 2 (HIV-1 and HIV-2) and Ebola virus. Simian foamy viruses (SFVs) are ancient retroviruses that infect Old and New World monkeys and apes. Although not known to cause disease, these viruses are of public health interest because they have the potential to infect humans and thus provide a more general indication of zoonotic exposure risks. Surprisingly, no information exists concerning the prevalence, geographic distribution, and genetic diversity of SFVs in wild-living monkeys and apes. Here, we report the first comprehensive survey of SFVcpz infection in free-ranging chimpanzees (*Pan troglodytes*) using newly developed, fecal-based assays. Chimpanzee fecal samples (n = 724) were collected at 25 field sites throughout equatorial Africa and tested for SFVcpz-specific antibodies (n = 706) or viral nucleic acids (n = 392). SFVcpz infection was documented at all field sites, with prevalence rates ranging from 44% to 100%. In two habituated communities, adult chimpanzees had significantly higher SFVcpz infection rates than infants and juveniles, indicating predominantly horizontal rather than vertical transmission routes. Some chimpanzees were co-infected with simian immunodeficiency virus (SIVcpz); however, there was no evidence that SFVcpz and SIVcpz were epidemiologically linked. SFVcpz nucleic acids were recovered from 177 fecal samples, all of which contained SFVcpz RNA and not DNA. Phylogenetic analysis of partial *gag* (616 bp), *pol*-RT (717 bp), and *pol*-IN (425 bp) sequences identified a diverse group of viruses, which could be subdivided into four distinct SFVcpz lineages according to their chimpanzee subspecies of origin. Within these lineages, there was evidence of frequent superinfection and viral recombination. One chimpanzee was infected by a foamy virus from a *Cercopithecus* monkey species, indicating cross-species transmission of SFVs in the wild. These data indicate that SFVcpz (i) is widely distributed among all chimpanzee subspecies; (ii) is shed in fecal samples as viral RNA; (iii) is transmitted predominantly by horizontal routes; (iv) is prone to superinfection and recombination; (v) has co-evolved with its natural host; and (vi) represents a sensitive marker of population structure that may be useful for chimpanzee taxonomy and conservation strategies.

## Author Summary

Cross-species transmissions of infectious agents from primates to humans have led to major disease outbreaks, with AIDS representing a particularly serious example. It has recently been shown that humans who hunt primates frequently acquire simian foamy virus (SFV) infections. Thus, these viruses have been proposed as an “early warning system” of human exposure to wild primates. In this study, we have tested this concept by developing non-invasive methods to determine the extent to which wild chimpanzees are infected with SFV. We analyzed more than 700 fecal samples from 25 chimpanzee communities across sub-Saharan Africa and obtained viral sequences from a large number of these. SFV was widespread among all chimpanzee subspecies, with infection rates ranging from 44% to 100%. The new viruses formed subspecies-specific lineages consistent with virus/host co-evolution. We also found mosaic sequences due to recombination, indicating that chimpanzees can be infected with multiple viral strains. One chimpanzee harbored an SFV from a monkey species, indicating cross-species transmission in the wild. These data indicate that chimpanzees represent a substantial natural reservoir of SFV. Thus, monitoring humans for these viruses should identify locations where human/chimpanzee encounters are most frequent, and where additional transmissions of chimpanzee pathogens should be anticipated.

## Introduction

Foamy viruses (also termed spumaviruses) are complex retroviruses that naturally infect numerous mammal species, including primates, felines, bovines and equines, but not humans [1]–[4]. Simian foamy viruses (SFVs) have been identified in a wide variety of primates, including prosimians, New World and Old World monkeys as well as apes, and each species has been shown to harbor a unique (species-specific) strain of SFV [5]–[13]. Moreover, closely related SFVs have been isolated from closely related primate species: a comparison of phylogenies derived from SFV integrase and primate mitochondrial DNA sequences revealed highly congruent relationships, indicating virus-host co-evolution for at least 30–40 million years [13]. This ancient relationship may be responsible for the non-pathogenic phenotype of SFV: Although highly cytopathic in tissue culture, the various SFVs do not seem to cause any recognizable disease in their natural hosts [2],[3],[14]. SFVs are highly prevalent in captive primate populations, with infection rates ranging from 70% to 100% in adult animals [2], [3], [5], [15]–[19]. Transmission is believed to occur through saliva because large quantities of viral RNA, indicative of SFV gene expression and replication, are present in cells of the oral mucosa [3], [20]–[22]. However, little is known about the prevalence and transmission patterns of SFV in wild-living primate populations.

Although there is no human counterpart of SFV, humans are susceptible to cross-species infection by foamy viruses from various primate species. Indeed, the first “human foamy virus” [23] isolated from a Kenyan patient with nasopharyngeal carcinoma more than three decades ago was subsequently identified to be of chimpanzee origin [7],[8]. Since then, SFV strains from African green monkeys, baboons, macaques and chimpanzees have been identified in zookeepers and animal caretakers who acquired these infections through occupational exposure to primates in captivity [19], [24]–[27]. More recently, about 1% of Cameroonian villagers who were exposed to primates through hunting, butchering and the keeping of pet monkeys were found to be SFV antibody positive, and genetic analysis of three such cases documented infection with SFV strains from DeBrazza's monkeys, mandrills and gorillas [10]. Finally, a large proportion of individuals (36%) who were severely bitten and injured while hunting wild chimpanzees and gorillas had detectable SFVcpz or SFVgor sequences in their blood [28]. Thus, humans are susceptible to a wide variety of SFVs and seem to acquire these viruses more readily than other retroviruses of primate origin, such as simian immunodeficiency viruses (SIVs) or simian T-lymphotropic viruses (STLVs). Interestingly, these infections appear to be non-pathogenic and thus far exhibit no evidence of onward transmission by human-to-human contact; however, additional studies will need to be conducted to fully characterize the natural history of SFV infections in humans [10], [24], [28]–[30].

**Figure 1:**
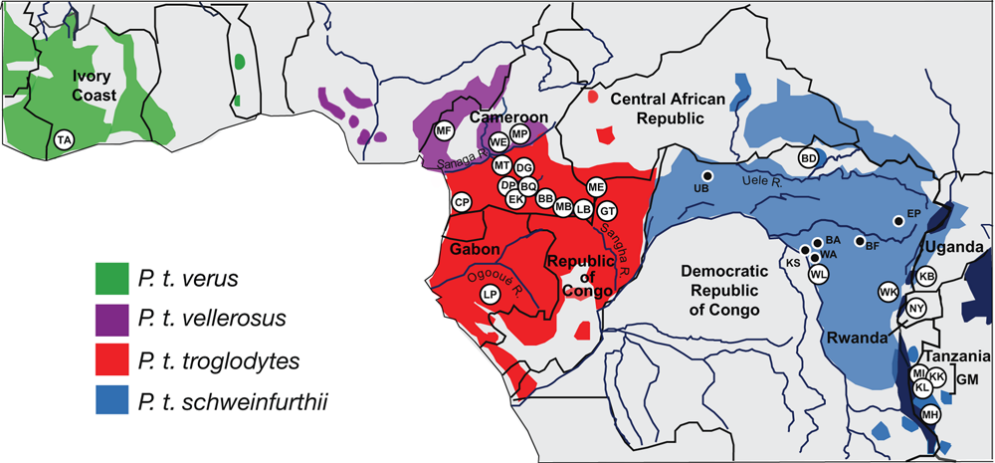
Location of wild chimpanzee study sites. Field sites are shown in relation to the ranges of the four proposed chimpanzee subspecies. White circles indicate forest areas where fecal samples were collected for prevalence studies (Table 3). These were located in Cote d'Ivoire (TA), Cameroon (MF, WE, MP, MT, DG, DP, BQ, CP, EK, BB, MB, LB), the Central African Republic (ME), Gabon (LP), Republic of Congo (GT), Democratic Republic of Congo (BD, WL, WK), Uganda (KB), Rwanda (NY), and Tanzania (GM-MT, GM-KK, GM-KL, MH). Black circles indicate forest sites where eight ancillary samples (BA432, BF1167, EP479, EP486, KS310, UB446, WA466, WA543) were collected. International borders and major rivers are shown.

Among wild primates, chimpanzees (*Pan troglodytes*) are of particular public health interest since they harbor SIVcpz, the precursor of the human immunodeficiency virus type 1 (HIV-1) [31]–[34]. There are four proposed chimpanzee subspecies which have been defined on the basis of geography and differences in mitochondrial DNA (mtDNA) sequences [35],[36]. These include *P. t. verus* in west Africa, *P. t. vellerosus* in Nigeria and northern Cameroon, *P. t. troglodytes* in southern Cameroon, Gabon, Equatorial Guinea and the Republic of Congo, and *P. t. schweinfurthii* in the Democratic Republic of Congo and countries to the east (Figure 1). Two of these, *P. t. troglodytes* and *P. t. schweinfurthii*, are naturally infected with SIVcpz, but only *P. t. troglodytes* apes have served as a reservoir of human infection [31]–[34]. It is now well established that SIVcpz*Ptt* has been transmitted to humans on at least three occasions, generating HIV-1 groups M, N and O. Moreover, two of these cross-species infections (groups M and N) have been traced to distinct *P. t. troglodytes* communities in southeastern and southcentral Cameroon, respectively [33]. The reason for the emergence of SIVcpz*Ptt* strains, but not SIVcpz*Pts* strains, in humans is unknown, but could reflect regional differences in the types and frequencies of human/chimpanzee encounters. Thus, examining humans for SFVcpz infection might be informative as to the location(s) where human/chimpanzee contacts are most common; however, no information exists regarding the prevalence, geographic distribution and genetic diversity of SFVcpz in chimpanzees in the wild.

In this study, we sought to develop an experimental strategy that would allow us to identify and molecularly characterize SFVcpz infection in wild-living chimpanzees by entirely non-invasive means. The rationale for this approach was two-fold. First, we wished to explore whether large scale screening of endangered primates for infectious agents other than primate lentiviruses was feasible. Second, we wished to examine whether SFVcpz could serve as a test case in efforts to develop suitable early warning systems for pathogens that might infect humans exposed to wild animals. To this end, we tested whether fecal based methods previously developed for SIVcpz could be adapted to the non-invasive detection and molecular characterization of SFVcpz. Our results show that this was indeed possible. Using these newly developed methods, we determined the prevalence of SFVcpz infection in wild chimpanzee communities throughout equatorial Africa, molecularly characterized 120 new SFVcpz strains, examined the subspecies association and phylogeography of SFVcpz, documented numerous instances of SFVcpz co-infection and recombination, investigated the routes of SFVcpz transmission in the wild, and examined the frequency of SFV cross-species transmissions from prey species. Our results reveal important new insights into the molecular ecology and natural history of SFVcpz infection that could not have been gained from studies of captive chimpanzees, and show more generally how endangered primates can be studied by non-invasive molecular approaches to elucidate the circumstances and mechanisms of pathogen transmission.

## Results

### Fecal-based methods for SFVcpz antibody and nucleic detection

SFV infection of primates and humans is generally diagnosed by documenting virus specific anti-Gag antibodies in serum or plasma using ELISA and/or Western blot approaches [8],[10],[18],[19]. The infecting SFV strain is then molecularly characterized by amplifying viral DNA from peripheral blood mononuclear cell (PBMC) or other tissue DNA [8], [10], [11], [13], [16]–[18],[24],[26],[28]. Since collecting blood from wild chimpanzees is not feasible, we sought to develop methods of SFVcpz detection that are entirely fecal-based. To accomplish this, we examined whether existing methods of SIVcpz fecal antibody and nucleic acid detection [33],[37],[38] could be adapted to the non-invasive identification and molecular characterization of SFVcpz.

**Figure 2:**
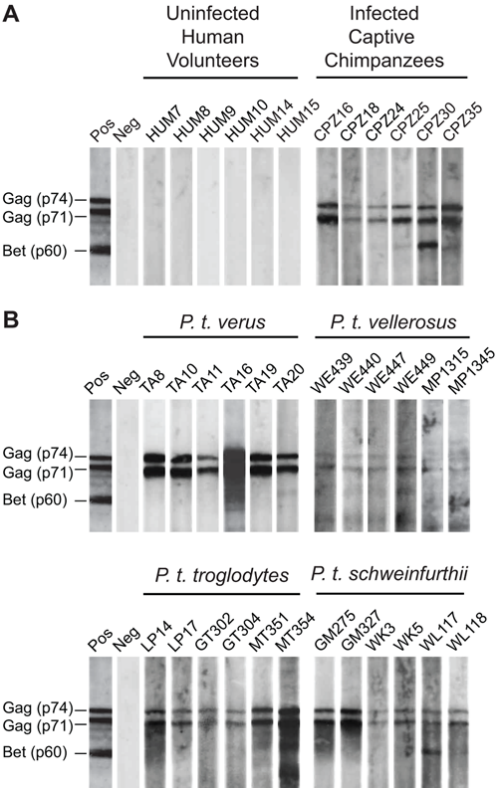
Detection of SFVcpz antibodies in chimpanzee fecal samples. Enhanced chemiluminescent (ECL) Western blot analysis of fecal extracts from (A) human volunteers and captive chimpanzees, and (B) SFVcpz infected wild chimpanzees representing four different chimpanzee subspecies. Strips were prepared using an infectious molecular clone (pMod-1) of SFVcpz*Pts* (see Methods). Samples are numbered, with letters indicating the species (panel A) or collection site (panel B) of origin. Molecular weights of SFVcpz specific Gag and Bet proteins are shown. The banding pattern of plasma from an SFVcpz infected chimpanzee (used at a 1∶100,000 dilution) and an uninfected human are shown as positive (Pos) and negative (Neg) controls, respectively.

Western blot strips were prepared from sucrose purified SFVcpz virions and used to test 40 fecal extracts from 23 SFVcpz infected chimpanzees from the Yerkes Primate Research Center (Table 1). Reactivity with the two SFVcpz Gag proteins p74 and p71 was scored positive, following interpretive guidelines established for serum antibody positivity [8],[10],[18]. The absence of viral bands was scored negative, and samples that did not meet either criterion were classified as indeterminant. Using this approach, SFVcpz specific IgG antibodies were detected in 29 of 40 fecal extracts from infected chimpanzees (Table 1). All samples reacted with the Gag doublet and a subset also recognized the accessory Bet (p60) protein (Figure 2A). In contrast, none of 21 fecal extracts from uninfected human volunteers exhibited false-positive or indeterminant Western blot reactivities (Figure 2A; Table 1).

**Table 1:** Validation of Fecal-Based Antibody and Nucleic Acid Detection Assays Using Samples from SFVcpz Infected Captive Chimpanzees and Uninfected Human Volunteers.

Captive chimpanzees^a^	Antibody positive samples/ number tested	vRNA positive samples/ number tested	vDNA positive samples/ number tested	SFVcpz strains^b^	Human Volunteers	Antibody positive samples/ number tested	vRNA positive samples/ number tested	vDNA positive samples/ number tested
CPZ 1	2/2	2/2	2/2	YK3	HUM 1	0/1	0/1	0/1
CPZ 2	1/1	1/1	0/1	YK3	HUM 2	0/1	0/1	0/1
CPZ 3	2/2	2/2	0/2	YK3	HUM 3	0/1	0/1	0/1
CPZ 4	2/2	1/2	0/2	YK5	HUM 4	0/1	0/1	nd^c^
CPZ 5	1/1	1/1	0/1	YK2	HUM 5	0/1	0/1	nd
CPZ 6	0/5	3/5	0/5	YK3	HUM 6	0/1	0/1	nd
CPZ 7	1/1	0/1	0/1	n/a^b^	HUM 7	0/1	0/1	nd
CPZ 8	3/3	1/3	0/3	YK18	HUM 8	0/1	0/1	nd
CPZ 9	1/1	1/1	0/1	YK5	HUM 9	0/1	0/1	nd
CPZ 10	1/1	1/1	0/1	YK15	HUM 10	0/1	0/1	nd
CPZ 11	1/1	1/1	0/1	YK18	HUM 11	0/1	0/1	nd
CPZ 12	2/2	2/2	0/2	YK26	HUM 12	0/1	0/1	nd
CPZ 13	4/4	4/4	0/4	YK22	HUM 13	0/1	0/1	nd
CPZ 14	1/1	1/1	0/1	YK23	HUM 14	0/1	0/1	nd
CPZ 15	1/1	1/1	0/1	YK29	HUM 15	0/1	0/1	nd
CPZ 16	2/2	2/2	0/2	YK30	HUM 16	0/1	0/1	nd
CPZ 17	0/1	1/1	0/1	YK32	HUM 17	0/1	0/1	nd
CPZ 18	2/2	2/2	0/2	YK15	HUM 18	0/1	0/1	nd
CPZ 19	1/1	1/1	0/1	YK15	HUM 19	0/1	0/1	nd
CPZ 20	1/2	0/2	0/2	n/a	HUM 20	0/1	0/1	nd
CPZ 21	0/1	0/1	0/1	n/a	HUM 21	0/1	0/1	nd
CPZ 22	0/1	0/1	0/1	n/a				
CPZ 23	0/2	2/2	0/2	YK3				
n = 23	29/40	30/40	2/40		n = 21	0/21	0/21	0/3

a All chimpanzees were housed at the Yerkes Primate Research Center; SFVcpz infection was confirmed by demonstrating virus specific (anti-Gag) antibodies in their blood.

The phylogenetic relationships of SFVcpz strains YK2-YK32 strains are shown in Figures 6–8.

b n/a, not available.

c nd, not done.

**Figure 3:**
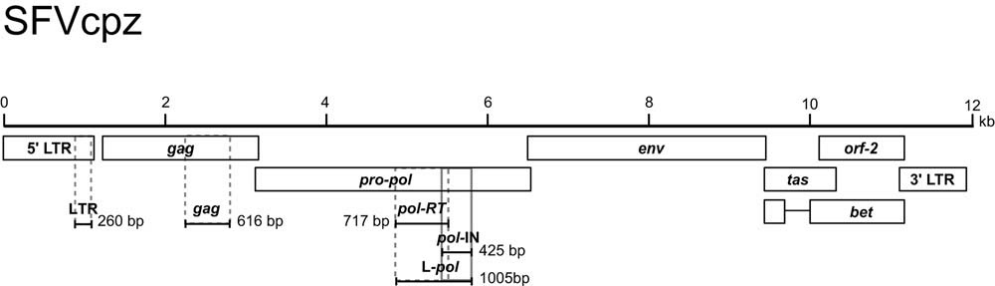
Location of RT-PCR derived amplicons in the SFVcpz genome. Amplification products are shown in relation to the corresponding regions in the SFVcpz genome, with the length of the amplified fragments indicated. The genomic organization of SFVcpz is shown on the top (structural and accessory genes are drawn to scale) [79].

We also investigated whether SFVcpz nucleic acids could be detected in fecal samples using primers designed to amplify a conserved 425 bp fragment (*pol*-IN) in the viral *pol* gene (Figure 3) [11]–[13],[18]. *In vitro* studies have shown that foamy viruses, in contrast to other retroviruses, reverse transcribe their RNA genome before they assemble into virus particles and bud from infected cells [39],[40]. Thus, infectious foamy virus particles have been reported to contain mostly viral DNA, while productively infected cells contain mostly viral RNA [1],[40],[41]. Using nested PCR to analyze fecal samples from the 21 infected chimpanzees, we found SFVcpz DNA in only 2 of 40 samples (Table 1). However, RT-PCR of fecal RNA from these same specimens yielded amplification products for 30 samples. Sequence analysis confirmed the authenticity of the amplification products and identified 11 distinct SFVcpz strains (Table 1). Omission of the cDNA synthesis step during the RT-PCR procedure failed to yield detectable amplification products. These results thus indicate that SFVcpz is present in chimpanzee fecal samples mostly as viral RNA, the source of which (cell associated, cell free, or both) remains to be determined.

The sensitivities of SFVcpz antibody and viral nucleic acid detection in fecal samples from captive chimpanzees were determined to be 73% and 75%, respectively (Table 2). Assay specificities were 100% (Table 1). Interestingly, not all fecal vRNA positive chimpanzees were also fecal Western blot positive (and vice versa). Two SFVcpz infected apes (CPZ6, CPZ23), each of whom had detectable RNA in at least two independent stool samples, were repeatedly fecal antibody negative (Table 1). Since both individuals had high titer antibodies in their blood, this was not due to a recently acquired SFVcpz infection. Two other apes (CPZ7, CPZ20) were fecal antibody positive, but virion RNA negative (Table 1). Thus, antibody or virion RNA screening alone would have missed SFVcpz infection in these individuals. Nonetheless, Western blot together with RT-PCR correctly diagnosed SFVcpz infection in 21 of 23 captive chimpanzees, suggesting that the newly developed assays were of sufficient sensitivity and specificity for field surveys, especially when used in combination.

**Table 2:** Sensitivities of Antibody and Viral RNA Detection in Fecal Samples From Captive and Wild Chimpanzees.

Sites^c^	Individuals^d^	SFVcpz Western blot^a^		SFVcpz RT-PCR^b^	
		Positive samples/ number tested	Sensitivity(95% CI)^e^	Positive samples/number tested	Sensitivity (95% CI)^e^
**Captive Apes**					
YK	23	29/40	0.73 (0.59–0.84)	30/40	0.75 (0.61–0.86)
**Wild-living Apes**					
TA	16	16/16	1.00 (0.83–1.00)	7/16	0.44 (0.23–0.67)
DP	24	20/62	0.32 (0.23–0.43)	19/28	0.68 (0.51–0.82)
EK	8	2/10	0.20 (0.04–0.51)	7/9	0.78 (0.45–0.96)
BB	10	1/13	0.08 (0.00–0.32)	10/10	1.00 (0.74–1.00)
MB	8	8/13	0.62 (0.35–0.83)	6/8	0.75 (0.40–0.95)
LB	4	1/8	0.13 (0.01–0.47)	4/5	0.80 (0.34–0.99)
GT	9	11/15	0.73 (0.49–0.90)	5/15	0.33 (0.14–0.58)
GM	26	43/51	0.84 (0.73–0.92)	16/32	0.50 (0.34–0.66)
MH	9	11/12	0.92 (0.66–1.00)	1/11	0.09 (0.00–0.36)

a Western blot strips were prepared using an infectious molecular clone (pMod-1) of SFVcpz*Pts* (see Methods).

b RT-PCR was performed using SFVcpz specific *pol*-IN primers.

c Sensitivities of SFVcpz antibody and viral RNA detection were determined for captive (YK) as well as wild-living chimpanzees at different field sites (TA, DP, EK, BB, MB, LB, GT, GM, MH).

d SFVcpz infection in captive chimpanzees was confirmed by demonstrating virus specific antibodies in their blood; SFVcpz infection of wild-living chimpanzees was determined by demonstrating virus specific antibodies or viral RNA in at least one fecal sample.

e The sensitivities of fecal antibody and viral RNA detection were calculated for each site based on the total number of samples collected from infected chimpanzees at that site (with 95% confidence intervals [CI]); the specificity of fecal antibody detection was determined by testing fecal samples from uninfected human volunteers (Table 1) and determined to be 1.00 (0.87–1.00); the specificity of virion RNA detection was set to 1.00 since all amplification products were sequence confirmed.

### Geographic distribution, subspecies association and prevalence of SFVcpz in wild-living chimpanzees

To determine to what extent chimpanzees are infected with SFVcpz in the wild, we tested 724 fecal samples from 25 different field sites across equatorial Africa for virus specific antibodies and/or viral RNA (Table 3). Samples were selected from existing specimen banks based on their geographic representation, available host genetic information (mtDNA, microsatellite and sex markers), and remaining quantities of material. Figure 1 depicts the geographic location of the sites with respect to the ranges of the four proposed chimpanzee subspecies. Except for *P. t. verus*, all other subspecies were sampled at multiple sites. Specimens from the Taï Forest (TA) as well as from Gombe (GM-MT, GM-KK) and Mahale Mountains (MH) National Parks were collected from individually known (habituated) chimpanzees under direct observation. Samples from the Goualougo Triangle (GT), several field sites in Cameroon (DP, EK, BB, MB, LB), and the Kalande community (GM-KL) in Gombe National Park were obtained from non-habituated chimpanzees, but were subsequently genotyped using mtDNA, microsatellite and sex markers and thus also represent known numbers of individuals [33] (B. Keele and B. H. Hahn, unpublished). Samples from the remaining field sites in Cameroon (MF, MP, WE, MT, BQ, DG, CP), Gabon (LP), the Central African Republic (ME), the Democratic Republic of Congo (BD, WL, WK), Rwanda (NY) and Uganda (KB) were derived from an unknown number of chimpanzees. All samples were previously screened for SIVcpz antibodies and/or vRNA [33],[37],[42] (F. van Heuverswyn and M. Peeters, unpublished) and their integrity was confirmed by mtDNA analysis (Table S1).

**Table 3:** Prevalence Rates of SFVcpz Infection in Wild Chimpanzees throughout Equatorial Africa.

Sites^a^	Subspecies^b^	Samples tested (WB/RT-PCR)^c^	Samples positive (WB/RT-PCR)^d^	Chimpanzees tested^e^	Chimpanzees infected	SFVcpz Prevalence^f^
TA	*P.t.v.*	16 (16/16)	16 (16/7)	16	16	100 (83–100)
MF	*P.t.vl.*	13 (13/13)	7 (0/7)	–h	–	98 (59–100)
MP	*P.t.vl.*	5 (5/5)	4 (2/4)	–	–	100 (22–100)
WE^g^	*P.t.vl.*	26 (26/13)	12 (8/9)	–	–	81 (55–97)
MT	*P.t.t.*	81 (81/14)	32 (32/7)	–	–	79 (65–88)
DG	*P.t.t.*	29 (29/29)	22 (4/22)	–	–	100 (81–100)
CP	*P.t.t.*	10 (10/8)	6 (1/6)	–	–	100 (55–100)
DP^i^	*P.t.t.*	114 (114/52)	34 (22/19)	45	24	60 (47–73)
BQ	*P.t.t.*	82 (82/21)	16 (9/10)	–	–	44 (31–58)
EK	*P.t.t.*	19 (19/15)	8 (2/7)	15	8	66 (41–85)
BB	*P.t.t.*	31 (31/18)	10 (1/10)	18	10	66 (44–84)
MB	*P.t.t.*	25 (25/16)	10 (8/6)	18	8	54 (33–74)
LB	*P.t.t.*	16 (16/8)	4 (1/4)	9	4	53 (23–81)
LP	*P.t.t.*	13 (10/12)	9 (6/4)	–	–	100 (61–100)
GT	*P.t.t.*	20 (20/20)	12 (11/5)	14	9	75 (50–90)
ME	*P.t.t.*	21 (21/21)	16 (0/16)	–	–	100 (74–100)
BD	*P.t.s.*	15 (15/15)	7 (2/7)	–	–	100 (65–100)
WL	*P.t.s.*	22 (20/5)	8 (8/1)	–	–	73 (44–93)
WK	*P.t.s.*	11 (10/4)	5 (4/3)	–	–	89 (44–100)
KB	*P.t.s.*	27 (27/15)	14 (14/2)	–	–	98 (78–100)
NY	*P.t.s.*	27 (27/18)	10 (6/4)	–	–	63 (37–85)
GM-KL	*P.t.s.*	30 (30/3)	23 (23/2)	14	9	85 (61–97)
GM-MT	*P.t.s.*	9 (6/7)	6 (4/2)	4	4	100 (47–100)
GM-KK	*P.t.s.*	42 (33/33)	24 (16/12)	25	13	64 (46–80)
MH	*P.t.s.*	20 (20/11)	11 (11/1)	17	9	75 (52–90)
n = 25		724 (706/392)	326 (211/177)	195	114	

a Location of sites is shown in Figure 1.

b *P.t.v.*, *P. t. verus*; *P.t.vl.*, *P. t. vellerosus*; *P.t.t.*, *P. t. troglodytes*; *P.t.s.*, *P. t. schweinfurthii*.

c Number of fecal samples tested for SFVcpz antibodies and/or viral RNA, with brackets indicating those tested by Western blot (WB) and those tested by RT-PCR, respectively.

d Number of fecal samples positive for SFVcpz antibodies and/or viral RNA, with brackets indicating those positive by WB and those positive by RT-PCR, respectively (the phylogenetic relationships of these newly derived SFVcpz strains are depicted in Figures 6–8).

e For four habituated communities (TA, GM-MT, GM-KK, MH) the number of tested chimpanzees was known; for seven non-habituated communities (GT, DP, EK, BB, MB, LB, GM-KL) the number of tested chimpanzees was determined by microsatellite analysis of fecal DNA [33].

f For sites where the number of chimpanzees was known, SFVcpz prevalence rates (%, with brackets indicating 95% confidence intervals) were estimated based on the proportion of infected individuals, taking into account the “field sensitivities” of the antibody and virion RNA detection tests. For sites where the number of chimpanzees was not known, prevalence rates were estimated based on the number of fecal samples tested, assuming that a fraction (17%) was partially degraded and that any given chimpanzee was sampled on average 1.72 times (see Methods for details).

g Based on mtDNA analysis, 24 samples were of *P. t. vellerosus* and 2 of *P. t. troglodytes* origin.

h –; not available.

i For this prevalence estimate, two WB indeterminant samples (reacting only with the Bet protein) were counted as negative.

Of 724 fecal samples included in the analysis (Table 3), 706 were tested by Western blot analysis and 211 were found to be SFVcpz antibody positive (18 samples were of insufficient quantity for immunoblot analysis but were used for RT-PCR amplification). All of these reacted with the Gag p74/p71 doublet and a small number also recognized the p60 Bet protein (Figure 2B). Interestingly, two samples from the DP site reacted only with the Bet protein and were thus classified as indeterminant (not shown). The remaining 493 fecal extracts exhibited no detectable bands and were thus classified as antibody (SFVcpz IgG) negative. A subset of samples (n = 392) was also examined for SFVcpz nucleic acids (Table 3). RT-PCR of fecal RNA yielded *pol*-IN (425 bp) amplification products for 175 samples, all of which were shown to contain SFVcpz sequences (two samples were RT-PCR positive using LTR and *pol*-RT primers, respectively). In contrast, amplification of fecal DNA from these same samples failed to yield viral sequences (not shown), providing further evidence for the presence of SFVcpz RNA, and not DNA, in fecal material. A breakdown of antibody and RNA positive samples for each field site is shown in Table 3. The results revealed SFVcpz infected chimpanzees at all field sites.

**Figure 4:**
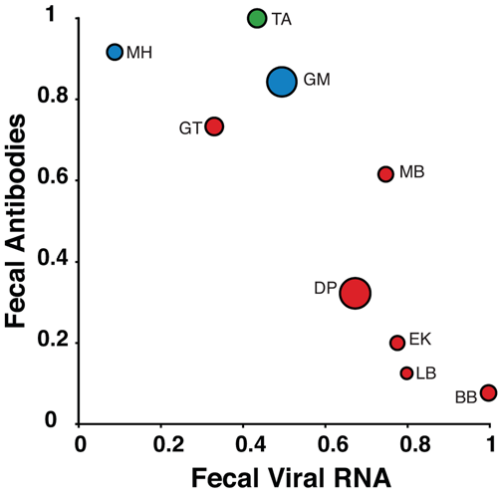
Inverse correlation of fecal antibody and viral RNA detection at different field sites. Fecal viral RNA (x-axis) and antibody (y-axis) detection sensitivities are plotted for field sites with known numbers of infected chimpanzees (Table 2). The size of the circle is directly proportional to the number of samples tested (results from the three Gombe communities were combined). Color coding and corresponding two letter codes are as in Figure 1. Test sensitivities are significantly inversely correlated (P<0.001).

We next sought to determine the prevalence of SFVcpz infection at each of the 25 field sites. To accomplish this, we examined whether fecal antibody and vRNA detection tests yielded similar data for captive as well as wild communities. Inspection of Table 3 indicated that this was not the case. For example, at the TA field site all of 16 fecal samples were SFVcpz antibody positive (100%), but only 7 contained vRNA (44%). In contrast, at the ME field site none of 21 fecal samples contained antibodies (0%), while 16 were vRNA positive (76%). Importantly, the latter was not due to a lack of antibody cross-reactivity since other *P. t. troglodytes* samples (e.g., 11 of 20 GT samples) were Western blot positive using the same antigens (Table 3, Figure 2B). To examine this further, we re-calculated test sensitivities using only samples from SFVcpz infected wild chimpanzees (Table 2). This yielded surprising results: not only did test results vary extensively between different field sites, the sensitivities of antibody and vRNA detection were also inversely correlated (Figure 4). To account for this in prevalence estimations, we decided to calculate a “field sensitivity” for each test by averaging values across all collection sites. The rationale for this was that the strong negative correlation between the two assay sensitivities would predict that if the sensitivity of one test was underestimated, the sensitivity of the other test would be overestimated to a roughly similar degree. Thus, if samples were subject to an equal number of both tests, these effects would tend to even out. While many samples were not subject to equal numbers of the two tests, this nonetheless seemed to represent the most reasonable approach. For both Western blot and RT-PCR assays, the average sensitivities across all sites were around 56%. Therefore we pooled results from all tests to obtain a general sensitivity value (56.3%) that was then used to calculate the prevalence rates.

Table 3 summarizes the prevalence of SFVcpz infection at 25 different field sites. For 11 sites, these values were calculated based on the proportion of infected individuals. For the remaining sites, prevalence rates were estimated based on the proportion of antibody and/or vRNA positive fecal samples, but correcting for repeat sampling and sample degradation (see Methods). The results revealed uniformly high infection rates for all sites, similar to previously reported values for captive primate populations [15]–[19]. The highest prevalences (>90%) were found at a *P. t. verus* field site in Cote d'Ivoire (TA); two *P. t. vellerosus* field sites in central Cameroon (MF, MP); four *P. t. troglodytes* field sites in Cameroon (DG, CP), Gabon (LP) and the Central African Republic (ME); and three *P. t. schweinfurthii* field sites in the DRC (BD), Uganda (KB) and Tanzania (GM-MT). The lowest infection rates (<60%) were identified at three *P. t. troglodytes* field sites in southern Cameroon (BQ, MB, LB). Given that the confidence intervals for the prevalence estimates across the various sites showed extensive overlap (Table 3), these differences are unlikely to be significant. These data thus indicate that SFVcpz is widely distributed and infects chimpanzees at very high prevalence rates throughout their natural habitat.

### Documentation of SFVcpz/SIVcpz co-infections

The fact that all 724 fecal samples had independently been tested for SIVcpz antibodies and/or viral nucleic acids provided an opportunity to compare the two viruses with respect to their relative infection frequencies and geographic distribution. As reported previously, natural SIVcpz infection has been identified only in two of the four chimpanzee subspecies (*P. t. troglodytes* and *P. t. schweinfurthii*), and then only in a fraction of sampled communities [33],[37],[42]. Thus, SIVcpz is clearly much less common and widespread among wild chimpanzees than is SFVcpz. Nonetheless, the current survey included field sites where both SFVcpz and SIVcpz infections were endemic. To examine whether the two infections were epidemiologically linked, we selected seven sites with known numbers of SFVcpz and/or SIVcpz infected chimpanzees. Four of these were located in Cameroon (MB, LB, EK, DP), while the other three were located in Gombe National Park in Tanzania (GM-KK, GM-KL, GM-MT). Table 4 summarizes the results: Of a total of 130 chimpanzees tested, 55 were infected only with SFVcpz, 7 were infected only with SIVcpz, and 15 harbored both viruses. Thus, SFVcpz/SIVcpz co-infections are not uncommon at locations where both viruses are endemic; however, examination of the relative frequencies of single and dual infections at individual sites, or sites in combination, revealed no association between SFVcpz and SIVcpz (Fisher exact test; P>0.2). Thus, there was no evidence that infection with one of these viruses increased or decreased the likelihood of infection by the other.

**Table 4:** Number of SFVcpz and SIVcpz Infections in Chimpanzee Communities Harboring Both Viruses.

Sites^a^	Chimpanzees tested	Infected only with SFVcpz	Infected only with SIVcpz	Co-infected with both SFVcpz and SIVcpz	Uninfected
DP	45	22	0	2	21
EK	15	6	2	2	5
MB	18	4	2	4	8
LB	9	3	1	1	4
GM-MT	4	3	0	1	0
GM-KK	25	12	1	1	11
GM-KL	14	5	1	4	4
n = 7	130	55	7	15	53

a Only collection sites with known numbers of infected individuals were included in this analysis.

### Patterns of SFVcpz transmission in the wild

**Figure 5:**
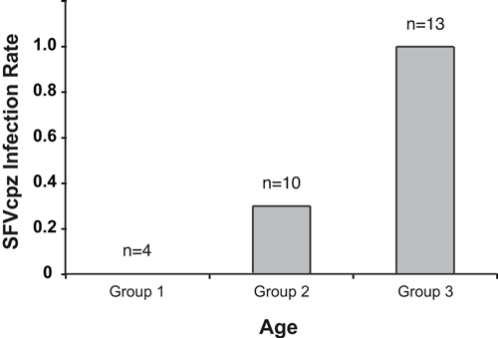
Increase of SFVcpz infection rates with age. Members of the habituated Mitumba and Kasekela communities in Gombe National Park were non-invasively tested for SFVcpz infection and their infection rate (y-axis) plotted by age group (x-axis). Group 1 comprises 4 infants age 2 or younger; group 2 comprises 10 chimpanzees age 2.1 to 9 years; and group 3 comprises 13 adult chimpanzees age 14 to 45.

To determine under what circumstances chimpanzees acquire SFVcpz in the wild, we screened members of two habituated communities for evidence of infection. The Kasekela and Mitumba communities are located in Gombe National Park and have been under human observation since the 1960s and 1980s, respectively [43]. Chimpanzees from both communities are followed daily (with particular individuals selected for all-day observation) and their reproductive states and social interactions are recorded. Thus, for many Mitumba and Kasekela apes, especially the more recent offspring, the date of birth is known. This provided an opportunity to compare the frequency of SFVcpz infection among individuals representing different age groups. Testing the most recent fecal sample available, we found no evidence of SFVcpz infection in four infants age 2 years or younger. In addition, only three of ten chimpanzees ages 2.1 to 9 years were found to be SFVcpz antibody and/or viral RNA positive. In contrast, all of 13 adult chimpanzees ages 14 to 45 years were SFVcpz infected (Figure 5). Thus, there was a significant increase of SFVcpz infection with age, suggesting horizontal rather than vertical (perinatal) transmission as the predominant route of infection in these communities.

To investigate whether perinatal transmission was responsible for at least some of the newly acquired infections, we tested longitudinal samples from the three SFVcpz positive offspring and their infected mothers. As shown in Table 5, Fansi (born in November 2001) was fecal Western blot positive in June 2004 (2.6 years of age), but both fecal antibody and viral RNA negative two years earlier in August of 2002. Similarly, Flirt (born in July 1998) was fecal Western blot positive in October 2001 (3.2 years of age), but antibody and viral RNA negative one year earlier in November 2000. Although false negative results at the earlier timepoints cannot be excluded, these data suggest that the two infants acquired SFVcpz after their first and third year of life, respectively. Analysis of the third mother/offspring pair also failed to provide evidence for perinatal transmission. Although Tarzan (born in October 1999) was SFVcpz fecal antibody positive at the earliest timepoint (2.6 years of age) and harbored a virus that was identical in its *pol*-IN sequence to that of his mother's, the same *pol*-IN sequences were also recovered from three other chimpanzees, including Flirt and one unknown individual from the neighboring Kalande community. Thus, it is unclear whether Tarzan acquired his SFVcpz infection from his mother during or shortly after birth, or whether he became infected later by another route. Taken together, none of these three mother/offspring pairs provided conclusive evidence for vertical transmission of SFVcpz in the wild.

**Table 5:** SFVcpz Infection in Three Mother-Offspring Pairs in Gombe National Park.

Individual^a^	Date of Birth	Age at Sampling (years)	SFVcpz infection		Relationship
			fecal antibodies	fecal vRNA	
Fansi	11/02/01	0.8	neg	neg	son
Fansi	11/02/01	2.6	pos	neg	son
Fansi	11/02/01	2.7	nd^b^	neg	son
Flossi	02/05/85	17.3	nd	neg	mother
Flossi	02/05/85	18.6	pos	neg	mother
Flossi	02/05/85	19.4	neg	*pol*-IN	mother
Flirt	07/20/98	2.4	neg	neg	daughter
Flirt	07/20/98	3.2	pos	neg	daughter
Flirt	07/20/98	3.8	nd	*pol*-IN	daughter
Fifi	07/02/58	44.6	pos	neg	mother
Fifi	07/02/58	45.1	neg	nd	mother
Fifi	07/02/58	45.2	pos	neg	mother
Tarzan	10/01/99	2.6	pos	neg	son
Tarzan	10/01/99	2.8	pos	*pol*-IN	son
Patti	07/02/61	40.5	nd	*pol*-IN,*gag*	mother
Patti	07/02/61	40.8	nd	*pol*-IN, *gag*, *pol*-RT	mother
Patti	07/02/61	42.1	pos	neg	mother
Patti	07/02/61	43.3	nd	*pol*-IN, *gag*	mother
Patti	07/02/61	43.5	nd	*pol*-IN, *gag*	mother

a All individuals were members of the Mitumba and Kasekela communities.

nd; not done.

### SFVcpz evolution at the subspecies level

**Figure 6:**
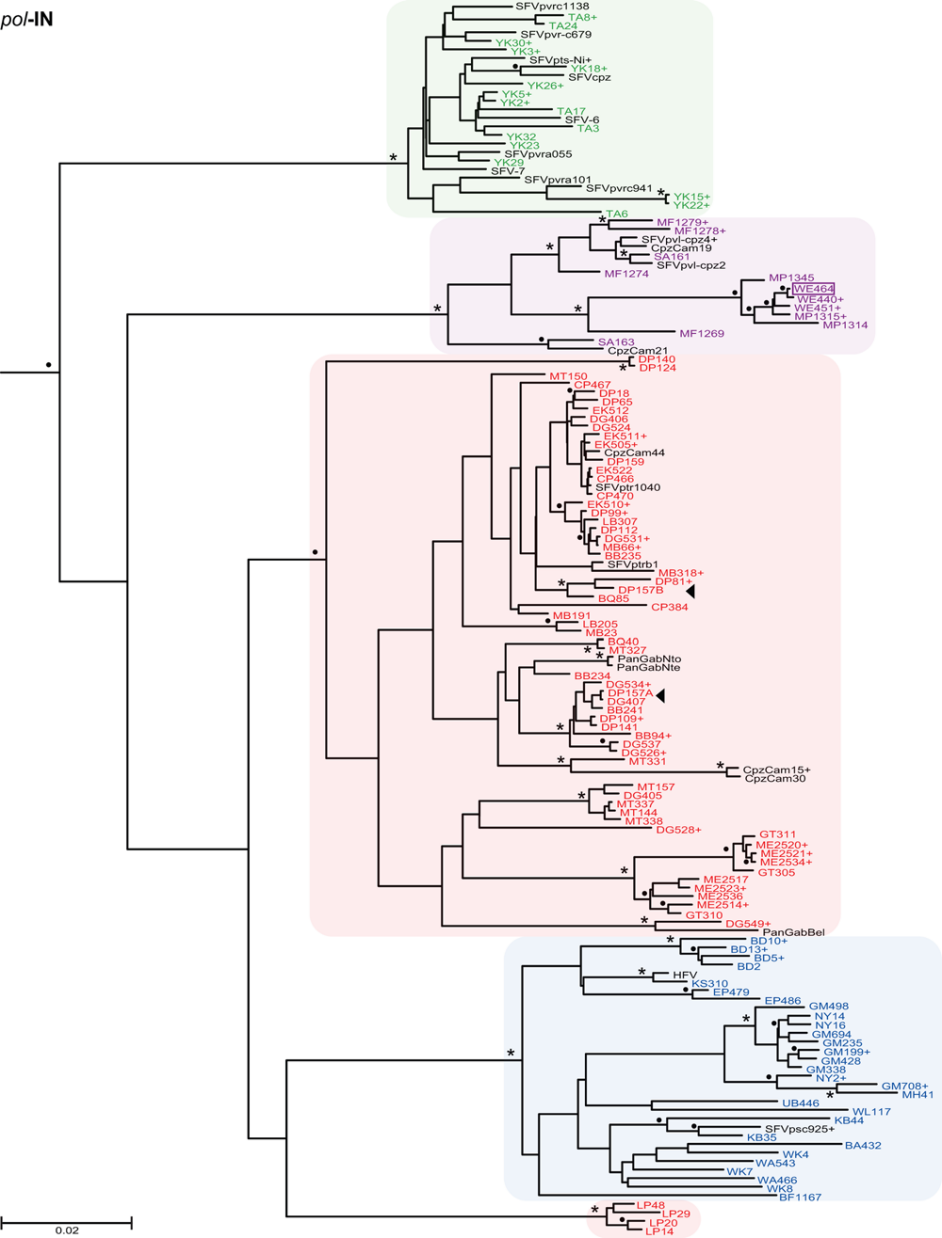
Evolutionary relationships of newly derived SFVcpz strains in the *pol*-IN region. *Pol*-IN (425 bp) sequences were analyzed using the Bayesian Markov chain Monte Carlo (BMCMC) method implemented in BEAST. Sequence LM183 (from a wild bonobo) was included as an outgroup. The maximum clade credibility (MCC) tree topology inferred using TreeAnnotator v1.4.7 is shown, with branch lengths depicting the mean value for that branch in the upper half of the MCMC sample. Posterior probabilities (expressed as percentages) are indicated on well-supported nodes, either as asterisks (100%) or filled circles (90%–99%). Newly identified SFVcpz strains are color coded according to their subspecies of origin (as shown in Figure 1). Representative strains from the database are shown in black. Plus signs (+) denote sequences that represent placeholders of multiple viruses with identical sequences (a complete list is provided in Table S2). Sample WE464 (boxed) was collected in the *P. t. vellerosus* range, but has a *P. t. troglodytes* mtDNA haplotype (Figure S1). Arrows identify distinct SFVcpz strains (termed A or B) that were found in the same sample. The scale bar represents 0.02 substitutions per site.

**Figure 7 ppat-1000097-g007:**
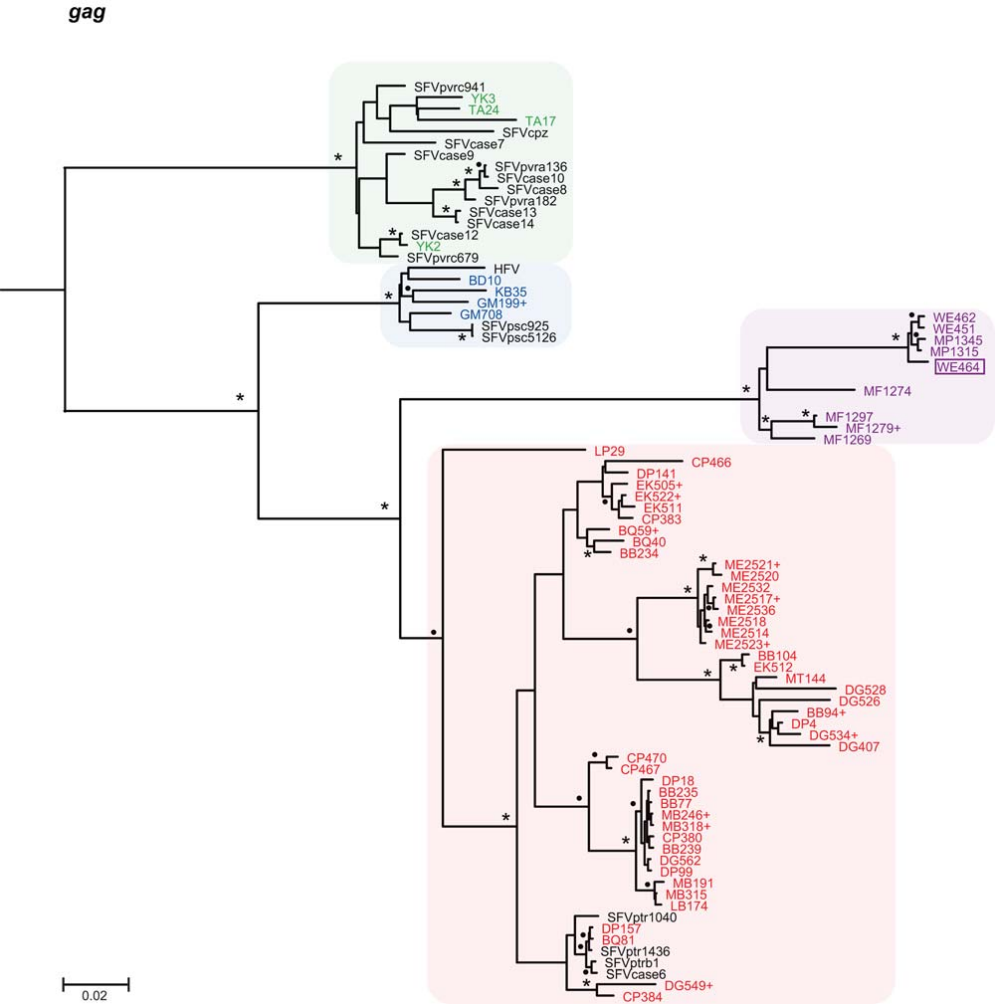
Evolutionary relationships of newly derived SFVcpz strains in the *gag* region. *Gag* (616 bp) sequences were analyzed as described in Figure 6. The *gag* tree was rooted using a relaxed clock. Posterior probabilities are indicated on well-supported nodes, either as asterisks (100%) or filled circles (90%–99%). Newly identified SFVcpz strains are color coded according to their subspecies of origin (Figure 1). Representative strains from the database are shown in black. Plus signs (+) denote sequences that represent placeholders of multiple viruses with identical sequences (Table S2). Sample WE464 (boxed) was collected in the *P. t. vellerosus* range, but has a *P. t. troglodytes* mtDNA haplotype (Figure S1). The scale bar represents 0.02 substitutions per site.

**Figure 8:**
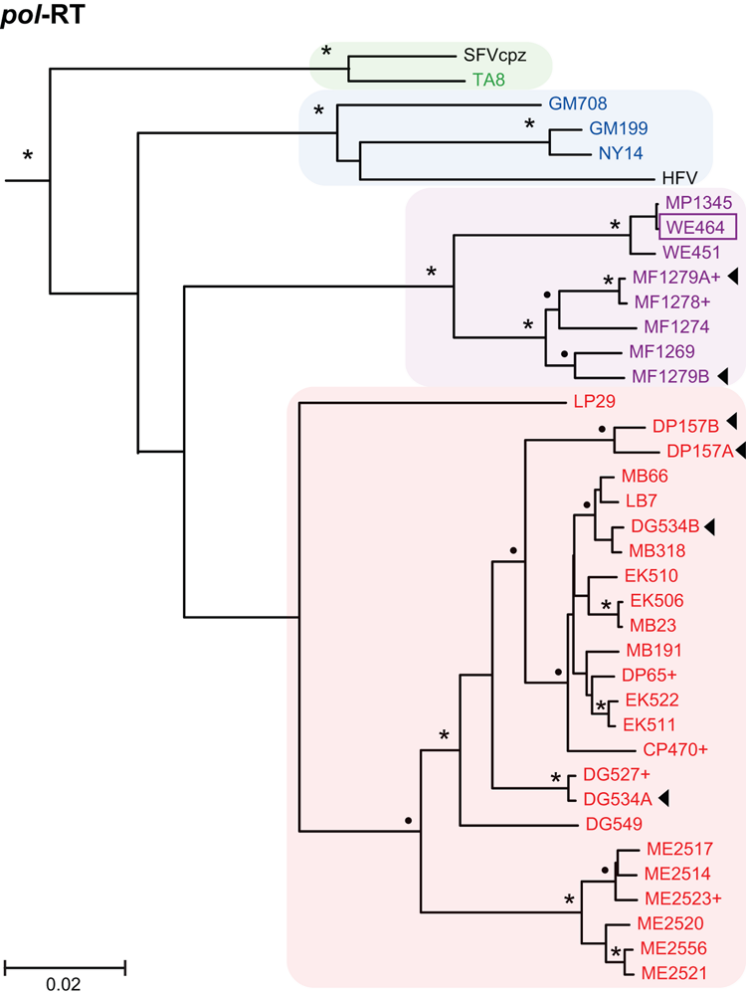
Evolutionary relationships of newly derived SFVcpz strains in the *pol*-RT region. *Pol*-RT (718 bp) sequences were analyzed as described in Figure 6. The tree was rooted using LM183 as an outgroup. Posterior probabilities are indicated on well-supported nodes, either as asterisks (100%) or filled circles (90%–99%). Newly identified SFVcpz strains are color coded according to their subspecies of origin (Figure 1). One representative strain from the database (HFV) is shown in black. Plus signs (+) denote sequences that represent placeholders of multiple viruses with identical sequences (Table S2). Sample WE464 (boxed) was collected in the *P. t. vellerosus* range, but has a *P. t. troglodytes* mtDNA haplotype (Figure S1). Arrows identify distinct SFVcpz strains (termed A or B) that were found in the same sample. The scale bar represents 0.02 substitutions per site.

To determine the evolutionary relationships of SFVcpz strains infecting wild chimpanzees in different parts of equatorial Africa, we selected 392 fecal samples for RT-PCR analysis. Using primers designed to amplify a conserved 425 bp *pol*-IN fragment [11]–[13],[18], we recovered SFVcpz sequences from 175 samples (one sample yielded only LTR and another only *pol*-RT sequences; not shown). *Pol*-IN sequences were also amplified from two *P. t. vellerosus* apes housed in a Cameroonian sanctuary (SA161 and SA163) as well as eight wild-living *P. t. schweinfurthii* apes who were sampled at different locations within the Democratic Republic of Congo (BA432, BF1167, EP479, EP486, KS310, UB446, WA466, WA543; Figure 1). The phylogenetic relationships of these SFVcpz sequences to each other and to subspecies specific SFVcpz reference sequences from the database are shown in Figure 6. The analysis revealed three well-defined SFVcpz clades for viruses from *P. t. verus*, *P. t. vellerosus*, and *P. t. schweinfurthii* apes, respectively, each supported with very high posterior probabilities. In contrast, SFVcpz strains from *P. t. troglodytes* formed two distinct (well-supported) groups in the maximum clade credibility (MCC) tree: (i) one major group which comprised the great majority of the newly identified *P. t. troglodytes* strains, and (ii) one minor group which included only four strains from the Lope Reserve in Gabon and which formed a sister clade to SFVcpz from *P. t. schweinfurthii* (Figure 6). Since the placement of the Lope group apart from the other *P. t. troglodytes* strains was not supported by a high posterior probability, we wondered whether its unexpected position in the MCC tree might be due to the short length of the *pol*-IN (425 bp) fragment. To clarify these relationships, we amplified additional *gag* (616 bp) and *pol*-RT (717 bp) fragments from a subset of samples. Indeed, phylogenetic analysis of these larger fragments placed a representative of the “Lope variant” (LP29) together with the other SFVcpz*Ptt* strains within a single cluster. In the *gag* region, this clade was supported with a highly significant posterior probability (Figure 7). In the *pol*-RT region, where the posterior probability was not high, the MCC tree nevertheless placed all *P. t. troglodytes* sequences in a monophyletic clade (Figure 8). Moreover, an analysis of combined *pol*-IN and *pol*-RT data (not shown) yielded a monophyletic *P. t. troglodytes* SFVcpz clade, with 100% posterior probability. Thus, SFVcpz strains from wild chimpanzees grouped into four major lineages according to their subspecies of origin.

To examine further the evolution of SFVcpz at the subspecies level, we obtained mitochondrial DNA sequences (hypervariable D loop region) from all SFVcpz vRNA positive fecal samples and performed a Bayesian Markov chain Monte Carlo (BMCMC) phylogenetic analysis (Figure S1). The topology of this tree was similar to previous mtDNA phylogenies in several key features [33]: (i) *P. t. verus* and *P. t. vellerosus* as well as *P. t. troglodytes* and *P. t. schweinfurthii* clustered together, forming two highly divergent lineages; (ii) *P. t. verus* and *P. t. vellerosus* formed two well separated sister clades; and (iii) *P. t. schweinfurthii* fell within the *P. t. troglodytes* radiation. Comparison of this mtDNA phylogeny with those of SFVcpz *pol*-IN, *pol*-RT and *gag* regions (Figures 6, 7, 8) revealed a number of differences. Most notably, SFVcpz strains from *P. t. vellerosus* were much more distant from SFVcpz strains infecting *P. t. verus* than would have been predicted based on mtDNA phylogenies of their respective hosts. In both *gag* and *pol*-RT trees, *P. t. vellerosus* viruses shared a most recent common ancestor with strains from *P. t. troglodytes* rather than with strains from *P. t. verus* (as with the placement of the Lope strains, the *gag* pattern was mirrored in the MCC tree from the *pol*-RT analysis, albeit without significant support). In addition, SFVcpz from *P. t. troglodytes* apes formed a single clade (Figures 7 and 8), while their corresponding mtDNA sequences were paraphyletic, being separated by the *P. t. schweinfurthii* clade (Figure S1). In many cases, chimpanzees with highly divergent mtDNA haplotypes harbored closely related SFVcpz strains, and *vice versa*. Finally, one fecal sample (WE464) collected north of the Sanaga River contained SFVcpz sequences from *P. t. vellerosus*, but mtDNA sequences from *P. t. troglodytes* (boxed in Figures 6, 7, 8, S1). While the latter finding is most simply explained by the migration of a *P. t. troglodytes* ape across the Sanaga River some time in the past, followed by infection of her progeny with the local variety of SFVcpz, the other discordances are more difficult to interpret. It is clear that SFVcpz is not strictly maternally inherited, since its evolutionary history shows differences with the mtDNA tree. Moreover, the mtDNA phylogeny (Figure S1) offers only a limited perspective on the ancestral relationships of chimpanzee populations, even setting aside any possible inaccuracies due to the short fragment analyzed. Thus, deciphering chimpanzee evolution in the more recent past will require additional study. However, the fact that 120 naturally occurring SFVcpz strains clustered in strict accordance with their mtDNA-defined subspecies of origin provides compelling evidence for virus-host co-evolution.

### Phylogeography of SFVcpz

As shown in Figure 1, three of the four chimpanzee subspecies were sampled at multiple locations. This provided an opportunity to examine whether viruses from *P. t. vellerosus*, *P. t. troglodytes* and *P. t. schweinfurthii* apes clustered according to their collection sites of origin, as previously reported for SIVcpz [33],[42]. Inspection of Figures 6–8 revealed that this was generally not the case. Although each of the major SFVcpz clades exhibited considerable structure, the great majority of sublineages were comprised of viruses from multiple field sites. Moreover, geographic distance did not predict viral diversity. For example, viruses from the single DG field site in southern Cameroon exhibited as much *pol*-IN inter-strain diversity (0% to 5.8%) as did viruses collected hundreds of kilometers apart at the CP and LB/MB field sites (0% to 4.1%). Nonetheless, there were some notable exceptions. Significant geographic clustering was observed for (i) *P. t. troglodytes* viruses from the ME and GT field sites in the Central African Republic and the Republic of Congo (Figures 6, 7, 8); (ii) *P. t. troglodytes* viruses from the LP field site in Gabon (Figure 6); and (iii) *P. t. schweinfurthii* viruses from the BD field site in the Democratic Republic of Congo (Figure 6). Interestingly, all of these were associated with potential barriers to chimpanzee movement. GT and ME were the only *P. t. troglodytes* field sites east of the Sangha River; LP was separated from all other *P. t. troglodytes* sites by the Ogooue River; and BD was the only *P. t. schweinfurthii* collection site north of the Uele River (Figure 1). Thus, in addition to delineating the subspecies ranges, major rivers and other biogeographical barriers appear to also have influenced the dispersal of SFVcpz within existing subspecies ranges.

### SFVcpz co-infection and recombination

GENECONV analyses and inspection of phylogenetic trees inferred for each independently amplified gene fragment (Figures 6, 7, 8) identified several SFVcpz strains with a strong signal of distinct evolutionary histories in different parts of their genome. For example, MF1269 was most closely related to other MF strains in *gag* and *pol*-RT regions (Figures 7 and 8), but clustered with MP and WE viruses in the *pol*-IN region (Figure 6). Such discordant branching patterns can be indicative of viral recombination but also of co-infection with divergent viruses [44]–[47]. Similarly, DG534, DP157, and CP470 were all found by GENECONV to be members of sequence pairs with globally significant (P<0.05) evidence of mosaic evolution. In the case of DG534 and DP157, highly significant putative recombination breakpoints were detected at or near the junction of independently amplified sequence fragments, strongly suggesting that some were due to the amplification of two or more variants from the same sample rather than intramolecular recombination *per se*.

**Figure 9:**
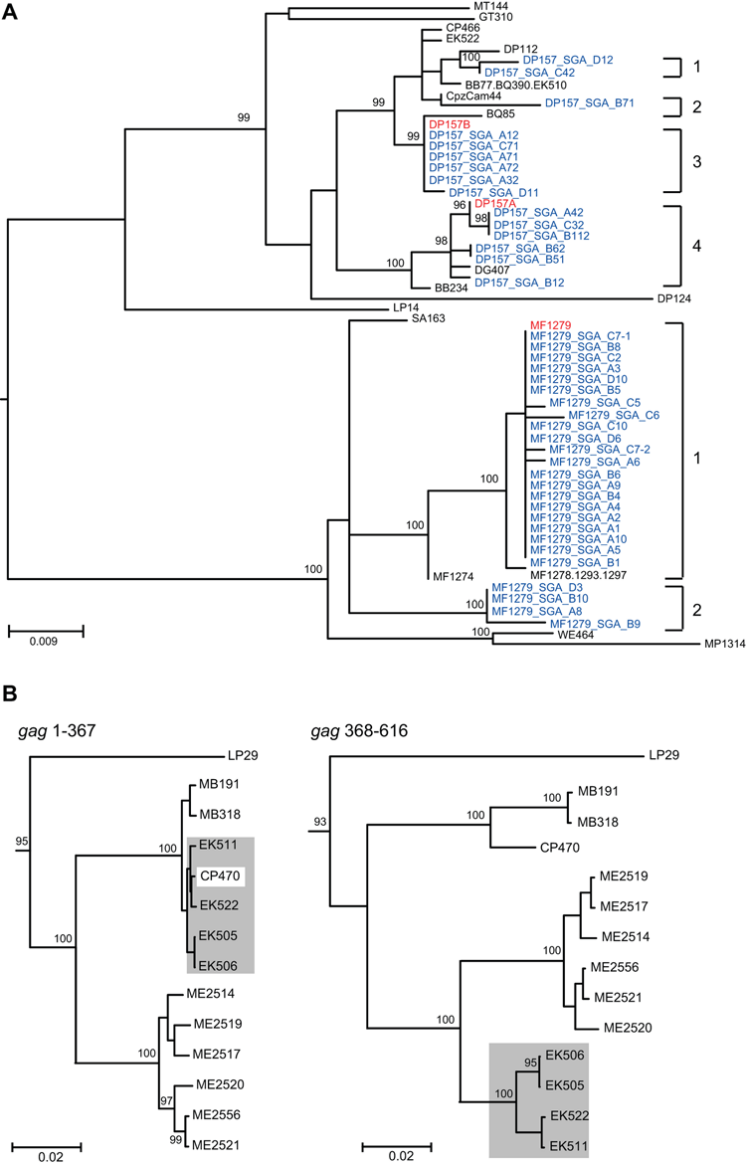
Coinfection and recombination in SFVcpz. (A) The maximum clade credibility (MCC) topology of *pol*-IN sequences is shown, with branch lengths as described in Figure 6. Brackets indicate the number of distinct SFVcpz strains that are present in samples DP157 and MF1279, respectively. Bulk PCR derived sequences are shown in red; SGA derived sequences are shown in blue. Numbers on nodes indicate posterior probabilities expressed as percentages (only values of 90% or higher are shown). The scale bar represents 0.009 substitutions per site. (B) Maximum clade credibility (MCC) topologies of *gag* sequences in two adjacent fragments are shown. Viruses that exhibit discordant branching patterns are highlighted. Numbers on nodes indicate percentage posterior probabilities. The scale bars represent 0.02 substitutions per site.

To differentiate between these possibilities, we selected five such samples (DG534, DP157, MF1279, MF1269, CP470) for additional RNA extraction, RT-PCR and sequence analyses. Comparison of independently amplified *gag*, *pol*-IN, and *pol*-RT sequences yielded unequivocal evidence of SFVcpz co-infection in three of the five fecal samples. As shown in Figures 6 and 8, DP157 harbored two clearly distinct variants (DP157A and DP157B). The observation that these sequences fall into different, highly supported clades (P = 1.0) within the *P. t. troglodytes* SFVcpz radiation leaves little doubt that this chimpanzee was co-infected with more than one strain. The alternative, i.e., that the divergent sequences trace back to a single infection that diversified extensively within a single chimpanzee, is inconsistent with the fact that sequences from other apes are interspersed between the DP157 variants. Similarly, DG534 and MF1279 each exhibited (at least) two distinct SFVcpz strains as determined by phylogenetic analysis of independently amplified (and directly sequenced) *pol*-RT sequences (Figure 8). To follow up on these observations, we subjected two of these samples (DP157 and MF1279) to single genome amplification (SGA). This approach amplifies single viral templates, precludes *Taq* polymerase errors and *in vitro* recombination, and provides an accurate representation of the viral population *in vivo* [48]–[50]. Targeting both *pol*-IN (Figure 9A) and *pol*-RT regions (not shown), we generated SGA derived sequences for MF1279 and DP157. Phylogenetic analysis of these sequences confirmed co-infection of MF1279 with two SFVcpz strains, and revealed the presence of at least four distinct SFVcpz strains in DP157 (Figure 9A).

In contrast, re-amplification experiments indicated that MF1269 and CP470 each harbored only a single identifiable virus. To determine whether MF1269 was truly mosaic, we first used GENECONV to examine its concatenated *gag*, *pol*-RT and *pol*-IN sequences for evidence of recombination. Pairwise analyses identified a potential recombination breakpoint in MF1269 near the *pol*-IN/*pol*-RT overlap (although this comparison fell marginally below significance according to the global P-value, which is corrected for multiple comparisons). We then amplified the corresponding *pol* fragment as a single genetic unit (Figure 3). The resulting L-*pol* sequence was identical to the concatenated MF1269 *pol*-IN and *pol*-RT sequences, indicating that the apparent signal of recombination could not be explained, in this case, by co-infection. Moreover, SGA amplification of the MF1269 L-*pol* fragment (which yielded four amplicons that differed from each other and the direct sequence by two or less nucleotide substitutions) excluded the possibility that the recombination breakpoint was a *Taq* polymerase induced PCR artifact [48]. Given the GENECONV evidence and more importantly, the 100% posterior probability support for MF1269 clustering on a different *P. t. vellerosus* SFVcpz lineage in *pol*-IN than in *gag* or *pol*-RT (Figures 6, 7, 8), we concluded that this sequence is a *bona fide* SFVcpz recombinant.

The CP470 case offered perhaps even stronger evidence of natural SFVcpz recombination. Having confirmed by repeated amplifications that this sample was not coinfected, we observed that the most parsimonious explanation for its inclusion by GENECONV in a sequence pair with a globally significant fragment (P<0.03) was that it was a “parent”, rather than a “daughter” (recombinant) sequence. GENECONV identified EK522 as the other sequence in the pair. Figure 9B indicates that it is this sequence and its close relatives (EK511, EK505, EK506) that appear to move from being closely related to CP470 (as well as MB191 and MB318) upstream of the identified breakpoint, to sharing a most recent common ancestor with the clade of ME viruses downstream of the breakpoint. We did not seek to reproduce the observed breakpoints in the EK clade because the fact that the sequences move across the tree together straightforwardly indicates that they evolved from a common recombinant ancestor.

It is worth noting that EK522 was the only one of this group with a globally significant P-value when compared with CP470; the other EK sequences all had highly significant pairwise P-values, but non-significant global values. Since they are all clearly closely related, this indicates that the global P-values represent a rather conservative measure of statistical significance for recombination in SFVcpz. It is thus highly likely that several of the numerous fragments that were significant in pairwise, but not global GENECONV comparisons also reflect recombination (data available upon request). Indeed, the strong phylogenetic evidence for recombination in MF1269 indicates that this is the case. Taken together, these results show for the first time that chimpanzees can be superinfected by different SFVcpz strains and that such superinfection can lead to recombination. They also suggest that recombination may occur rather frequently in SFVcpz.

### Cross-species transmission of SFV

**Figure 10:**
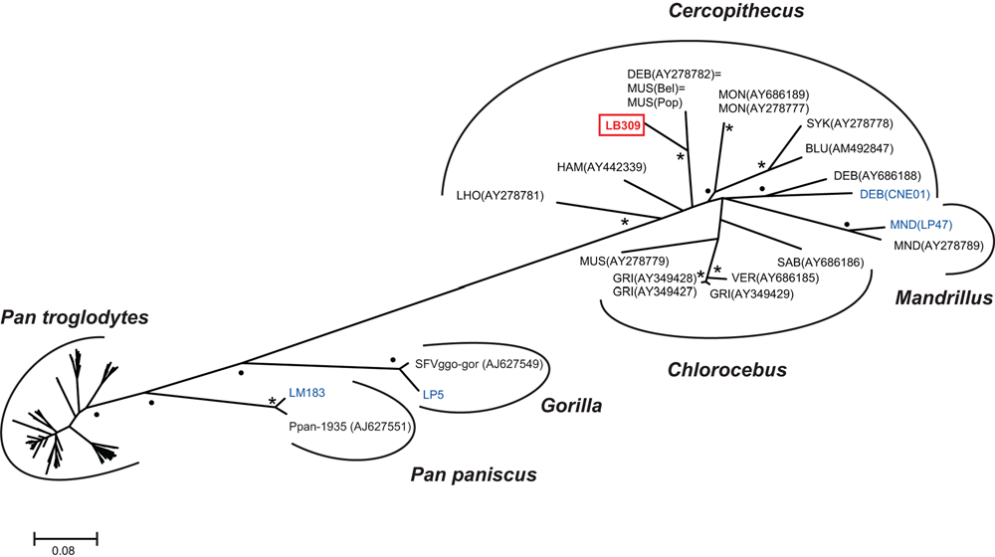
Cross-species transmission of SFV in the wild. The maximum clade credibility (MCC) topology of *pol*-IN sequences is shown, with branch lengths as described in Figure 6. The chimpanzee SFV strain LB309 (red box) significantly clusters within a group of SFVs previously derived from captive L'Hoest's (LHO), Hamlyn's (HAM), mustached (MUS), DeBrazza's (DEB), mona (MON), Sykes's (SYK) and blue (BLU) monkeys (GenBank accession numbers are indicated in parentheses), thus strongly suggesting a *Cercopithecus* monkey origin. Newly derived SFV sequences from a bonobo (LM183), gorilla (LP5), mandrill (LP47) and DeBrazza's monkey (CNE01) are also shown (blue) in relation to reference sequences from *Chlorocebus* and *Mandrillus* species (black). Numbers on nodes indicate posterior probabilities expressed as percentages (only 90% or higher are shown). The scale bar represents 0.08 substitutions per site.

Chimpanzees are avid hunters and frequently prey on smaller monkeys [51]–[53]. Since exposure to primates through hunting promotes acquisition of SFV by humans [10],[28], we wondered whether this was also the case in chimpanzees. Using conserved *pol*-IN primers previously shown to amplify divergent SFV strains [11]–[13],[18], we uncovered one simian foamy virus that did not fall within the SFVcpz radiation (Figure 10). This virus, termed LB309, was identified in a male member of a group of nine *P. t. troglodytes* apes sampled at the LB field site [33]. Phylogenetic analysis indicated that this unusual virus was most closely related to SFV strains previously identified in captive DeBrazza's (*Cercopithecus neglectus*) and mustached (*Cercopithecus cephus*) monkeys housed in an African primate facility (W. Switzer, unpublished). Since LB309 was identified in only a single fecal sample, we considered the possibility that it represented a mixture of chimpanzee and monkey fecal material; however, the fact that previous host genetic studies had yielded unambiguous microsatellite, sex and mtDNA data (Table S2 in [33]) rendered this scenario highly unlikely. We also looked for co-infection with chimpanzee foamy virus since four other chimpanzees from the LB site harbored SFVcpz (Table 3; Figure 6); however, repeated failure to amplify SFVcpz specific *gag* and *pol*-RT sequences suggested LB309 was the only (productively) infecting SFV strain. Although the species origin of LB309 could not be determined, this represents the first documented case of a monkey-to-ape transmission of SFV in wild *P. t. troglodytes* apes.

## Discussion

A primary objective of this study was to explore whether non-invasive detection methods previously developed for SIV could be adapted to the identification of other infectious agents in endangered primates. We selected SFV infection of wild chimpanzees as a test case for several reasons: First, SFVs can infect humans who come in contact with primates and may thus represent suitable markers of human zoonotic exposure risks [8], [10], [13], [19], [24]–[28]. Given that chimpanzees are naturally infected with several known human pathogens [33],[54],[55], determining the prevalence and genetic diversity of SFVcpz represented a first step toward examining the utility of this virus as a sentinel for human zoonoses. Second, although seemingly non-pathogenic in natural and non-natural hosts, SFVs could alter the course of SIV and HIV infections since dual SFV/HIV infections have been documented both in sex worker and blood donor cohorts in Africa [22],[56]. Thus, screening chimpanzees for both infections provided an opportunity to examine whether SIVcpz and SFVcpz are epidemiologically linked. Finally, foamy viruses are being explored as vaccine and gene therapy vectors for various human diseases [57]–[59]. It thus seemed prudent to study at least one member of this virus group in its natural host. To this end, we developed new SFVcpz specific fecal detection methods and used these to conduct a large-scale molecular epidemiological survey of wild chimpanzees throughout equatorial Africa. Our results indicate that non-invasive screening strategies can be extended to other infectious agents and show more generally how endangered primates can be studied by non-invasive molecular approaches.

Although both SIVcpz and SFVcpz infected chimpanzees secrete antibodies and nucleic acids into their feces, we found marked differences in the detection sensitivities of these viral markers between the two infections. The most striking difference was the extreme variability with which fecal antibodies and/or viral RNA were detected in SFVcpz infected apes from different communities (Table 2). For example, at the TA and MH field sites nearly all SFVcpz infected chimpanzees were antibody positive (Western blot sensitivities of 100% and 92%, respectively), but only very few had detectable viral RNA in their feces (44% and 9%, respectively). In contrast, at the BB and LB field sites nearly all SFVcpz infected chimpanzees were vRNA positive (100% and 80%, respectively), but only very few had detectable antibodies in their feces (8% and 13%, respectively). A comparison of test sensitivities across all field sites indicated that these values were inversely correlated (Figure 4). To determine whether this was due to a technical artifact, we re-analyzed nearly 300 antibody negative samples (including 74 specimens containing SFVcpz RNA) using newly produced Western blot strips and freshly prepared fecal extracts. Except for 17 weakly reactive samples, all others remained antibody negative. We also analyzed 34 IgG negative fecal samples for the presence of SFVcpz specific IgA. None of these were positive, consistent with the absence of SFVcpz specific IgA in other chimpanzee mucosal compartments [60]. Finally, we repeated RT-PCR analysis on a select number of RNA negative samples, but failed to uncover new SFVcpz sequences. Thus, the observed differences in fecal antibody and vRNA detection sensitivities cannot be explained by uneven test performance. Instead, SFVcpz infected chimpanzees appear to shed virus specific antibodies and nucleic acids only intermittently. Whether these fluctuations reflect true temporal differences in fecal antibody secretion and virus replication, or are the consequence of generally lower production levels that sometimes fall below the limits of detection, will require further study. However, in light of the data in Figure 9, it is tempting to speculate that the observed inverse correlations reflect, at least in part, different stages of recurring SFVcpz superinfection cycles where high titer viral replication at mucosal sites elicits an effective humoral (and possibly also cellular) immune response which reduces fecal viral load until the next infection cycle ensues. Regardless of the underlying mechanism(s), the observed fecal antibody and viral RNA fluctuations are in stark contrast to chronic SIVcpz infection where fecal antibodies are detected at all times with high sensitivity (92%), and where vRNA is amplified from virtually all antibody positive (non-degraded) fecal samples especially when different PCR primer sets are used [33],[42]. Thus, a screening algorithm consisting of an initial fecal antibody test followed by RT-PCR of only antibody positive samples (which is the standard approach for non-invasive SIVcpz surveys) is clearly not suitable for molecular epidemiological studies of SFVcpz. Instead, reliable non-invasive SFVcpz prevalence estimates require the use of both vRNA and antibody detection tests.

SFV infection is latent in most tissues, except for lung and tissues of the oral pharynx which express large quantities of viral RNA (up to 10^4^ copies per cell) and thus represent primary sites of SFV replication [20]–[22]. SFV replication has also been observed in the mesenteric lymph nodes and small intestine of SIVmac infected macaques [22]; however, even in these severely immune compromised animals, there was no evidence of SFV replication in the large intestine [22]. In light of these data, our finding of SFVcpz RNA in a large number of fecal samples comes as a surprise. Passage through the stomach would be expected to degrade both cell and virion associated SFVcpz RNA. It is thus highly unlikely that the fecal RNA that we observe is produced in the oral mucosa. Instead, it seems more likely that gut epithelial cells represent a primary site of SFV replication, at least at some stage during natural infection. Given the apparent fluctuations in fecal RNA shedding, it is easy to envision how this could have previously gone unrecognized [22]. We did not determine the copy number of SFVcpz RNA in the feces and thus cannot estimate how many cell equivalents are required to account for the detected amounts. However, in addition to SFVcpz, we also amplified SFV RNA from a limited number of bonobo, gorilla and mandrill stool samples, all of which were collected in the wild (Figure 10). It is thus clear that fecal RNA shedding is a common property of this entire group of viruses. It will be interesting to determine whether SFV RNA containing stool samples are infectious. This could explain why some zoo workers and animal handlers who never had direct physical contact with non-human primates were found to be SFV infected [8],[19].

In addition to its production site, the source of the SFVcpz RNA in stool samples remains a mystery. Unlike in other retroviruses, reverse transcription of the SFV genome takes place during budding and virion assembly, resulting in the production of SFV particles that contain both viral DNA and RNA [39],[40]. The viral RNA that we detect may thus derive from cell free virions and/or from mRNA and genomic RNA present in productively infected cells that are sloughed off into the feces. However, since SFV particles often bud at intracellular membranes [61], we would expect to also detect viral DNA. Instead, we found SFVcpz DNA in only 2 of 40 fecal samples from captive chimpanzees, and in none of 173 samples (including 87 SFVcpz RNA positive specimen) from wild chimpanzees. Thus, it remains unknown whether the SFVcpz RNA present in fecal samples is cell-derived, particle-derived, or a combination of both. Given our findings, it may also be of interest to determine whether currently used *in vitro* culture systems accurately reflect SFV replication *in vivo*.

Our survey of 25 different chimpanzee communities revealed high prevalence rates of SFVcpz infection across equatorial Africa. This observation, together with the lack of geographic clustering of most SFVcpz strains, and the obvious propensity of SFVcpz to superinfection and recombination, indicates that SFVcpz is a highly transmissible virus. Previous studies have indicated horizontal routes as the primary mode of SFV transmission [2],[17],[62]. Our findings in Gombe National Park are consistent with these observations. The fact that we detected SFVcpz in each of 13 adult chimpanzees, but in only 3 of 14 infants and juveniles indicates a clear increase of SFVcpz prevalence with age. In addition, we found no conclusive evidence for perinatal transmission. Two of the three infected offspring were SFVcpz negative at the time of first analysis, and the third one harbored a virus that was genetically indistinguishable in the *pol*-IN region from viruses infecting unrelated chimpanzees. Thus, perinatal transmission of SFVcpz, if it occurs at all, appears to be uncommon in wild-living chimpanzees. Instead, chimpanzees appear to acquire SFVcpz by horizontal routes, most likely by exposure to saliva (or feces), as has been proposed for other primates [17],[20],[62]. Indeed, young chimpanzees stay with their mothers until they are 8 or 9 years old and often share food. Thus, infants and juveniles are frequently exposed to their mother's saliva, which may constitute a common source of infection. In contrast, SIVcpz appears to be transmitted primarily by sexual (and sometimes perinatal) routes ([37]; Keele et al., unpublished). In light of these differences, the absence of an epidemiological link between SIVcpz and SFVcpz infections is perhaps not too surprising. Examining seven different communities, we found no indication that infection with one of these viruses increased or decreased the likelihood of infection by the other.

Simian foamy viruses are believed to have co-evolved with their respective primate hosts for millions of years [13], and our finding of subspecies-specific SFVcpz lineages is consistent with this hypothesis. Remarkably, all of the 120 newly characterized SFVcpz strains clustered according to their subspecies of origin. This included one strain from a site (WE) just north of the Sanaga River (i.e., within the range of *P. t. vellerosus*) infecting an individual with *P. t. troglodytes* mtDNA, indicating gene flow, but not viral flow, across a subspecies boundary. This monophyly of SFVcpz strains from each subspecies contrasts with the mtDNA phylogeny where *P. t. schweinfurthii* sequences lie within the *P. t. troglodytes* radiation. While the validity of classifying chimpanzees into subspecies has been questioned [63], the SFVcpz phylogeny corroborates the existence of four geographically isolated chimpanzee populations and the absence of SFVcpz transmission between subspecies argues that they are effectively separated, especially since such transmissions are frequently observed in captive settings (e.g., see DEB and MUS SFVs in Figure 10). The SFVcpz and mtDNA phylogenies (Figures 6, 7, 8, S1) differed with regard to the relationships among the four subspecies. However, these differences do not undermine the co-evolution hypothesis. When successive speciation events occur over a relatively short timescale, persistence of polymorphism from one event to the next means that any one genetic marker may not have the same phylogeny as the species [64]; this phenomenon is even more likely with recent subspeciation events. Thus, even if there has been complete co-evolution of SFVcpz with chimpanzees, discordance between the SFVcpz and mtDNA phylogenies may appear because either, or both, differ from the true historical relationships among the subspecies. In fact, the apparently shorter coalescence time of SFVcpz indicated by the reciprocal monophyly of *P. t. troglodytes* and *P. t. schweinfurthii* viruses suggests that SFVcpz could be less susceptible to this problem than mtDNA. Thus, SFVcpz may emerge as a more sensitive marker of population structure that may be useful for chimpanzee systematics as well as conservation strategies.

Phylogenetic analyses identified discordant branching orders for several SFVcpz strains, suggesting co-infection or recombination [44]–[47]. To examine whether this was indeed the case, we selected a subset of samples for repeat RT-PCR analyses, including single genome amplification (SGA) of re-extracted fecal viral RNA. SGA amplifies single viral templates, is not subject to *Taq* polymerase induced nucleotide substitutions and recombination, and thus provides an accurate representation of the viral population in the individual [48]–[50]. Adapting this approach to fecal RNA provided new insights into SFVcpz biology. SGA analysis formally documented infection with more than one virus in two chimpanzees. One of these apes (MF1279) was infected with two distinct SFVcpz strains, while the other (DP157) harbored at least four genetically diverse viruses. In both cases, predominant viral forms were identified by bulk RT-PCR (red in Figure 9A), but SGA was required to characterize the full extent of viral diversity in the sample, including the relative proportion of different variants. Repeat RT-PCR and SGA analyses also documented mosaic genome structures in several SFVcpz strains and demonstrated that these did not represent PCR artifacts. Although preliminary, these results suggest that superinfection and recombination occur rather frequently. As mentioned above, successive superinfection cycles may account at least for some of the observed fluctuations in fecal antibody and viral RNA detection in different chimpanzee communities. It will be interesting to test this hypothesis in chimpanzees from Gombe National Park where longitudinal samples from SFVcpz infected apes are available.

Because they are avid hunters, chimpanzees are also frequently exposed to SFV strains from other primate species. Testing 392 fecal samples for SFVcpz viral RNA, we found one male chimpanzee to harbor an SFV strain (LB309) that was closely related to viruses previously identified in captive DeBrazza's and mustached monkeys (Figure 10). The finding of LB309 RNA indicated a productive viral infection in the chimpanzee host. Similar findings were recently reported for chimpanzees from the Taï Forest where 3 of 12 apes studied harbored SFV strains from sympatric western red colobus monkeys [65]. Interestingly, these apes (all males) were also coinfected with SFVcpz; however, it was not determined whether the dual infections were productive since viral DNA (and not RNA) sequences were amplified from spleen necropsy specimens using strain specific PCR primers [65]. Since we did not use strain specific primers, it is likely that our data grossly underestimate the frequency of SFV cross-species transmission in the wild. Moreover, the failure of these cross-species infections to initiate secondary spread suggests that their replication (and thus fecal detection) may be limited. However, the examples demonstrate that chimpanzees, like humans, are susceptible to SFVs from other primate species, and the fact that all cross-infected apes were males (who hunt more frequently and eat more meat than females) strongly suggest that these transmissions occur in the context of predation. These findings may be of use to primatologists interested in chimpanzee hunting behavior and prey preferences in the wild.

Finally, SFVs are of public health interest because people in sub-Saharan Africa are routinely exposed to these viruses in the context of primate bushmeat hunting [10],[28]. We show herein that SFVcpz infection is highly prevalent in wild chimpanzee populations throughout their natural range. Thus, monitoring humans for SFVcpz infection should be informative as to the locations where human/chimpanzee encounters are most frequent and where additional cross-species transmissions should be anticipated. One such area is southern Cameroon where chimpanzees are endemically infected with SIVcpz strains that have already crossed the species barrier to humans, in one case (HIV-1 group M) with devastating consequences [33]. Screening humans for SFVcpz infection may also provide new insight into the environmental circumstances that underlie cross-species transmissions. For example, if the frequency of human SFVcpz infection were significantly lower in east compared to west central Africa, this would argue for lower exposure rates and, in turn, provide a reason why SIVcpz strains from *P. t. schweinfurthii* apes have not emerged as human pathogens. Thus, human SFVcpz infection should be formally investigated a sentinel for ape-derived pathogens, including new SIVcpz/HIV-1 outbreaks.

## Methods

### Captive chimpanzees

Fecal samples (n = 40) were collected from 23 captive chimpanzees housed at the Yerkes National Primate Research Center (Table 1), all of whom were known to be chronically infected with SFVcpz [8],[66]. Fecal samples were also obtained from nine *P. t. vellerosus* apes housed in a Cameroonian sanctuary (SA) who were of unknown SFVcpz infection status. Samples were preserved in RNA*later*, shipped and processed as described [33],[37]. All studies were carried out in strict accordance with international guidelines for the ethical scientific use and humane care of primates in research (the Yerkes National Primate Research Center is fully accredited by the Association for Assessment and Accreditation of Laboratory Animal Care International).

### Human volunteers

Blood and fecal samples were collected from 21 human volunteers who had no previous contact with primates or primate tissues (informed consent was obtained and the study protocol was approved by the University of Alabama at Birmingham Committee for Human Research). All of these individuals were seronegative for SFVcpz antibodies as determined by Western blot analysis (Table 1).

### Wild chimpanzees

The fecal samples (n = 732) used in this study were selected from existing banks of specimens previously collected for molecular epidemiological studies of SIVcpz [33],[37],[67],[68]. The great majority (n = 724) were collected from chimpanzee communities in Cote d'Ivoire, Cameroon, the Central African Republic (CAR), Gabon, the Republic of Congo (RC), the Democratic Republic of Congo (DRC), Uganda, Rwanda, and Tanzania (white circles in Figure 1). Eight additional samples (BA432, BF1167, EP479, EP486, KS310, UB446, WA466, WA543) were collected at various locations in the DRC (black circles in Figure 1). All samples were collected in the wild, and their species and subspecies origin were confirmed by mitochondrial DNA analysis. All samples were also screened for SIVcpz antibodies and/or nucleic acids. Of the 732 samples, 87 were collected from habituated chimpanzees under direct observation. These included members of the North and South communities in the Taï Forest (TA), Cote d'Ivoire [69], the Mitumba and Kasekela communities in Gombe National Park (GM-MT, GM-KK), Tanzania [43],[70], and the M group in Mahale Mountains National Park (MH), Tanzania [71]. At seven additional field sites, the number of sampled individuals was retrospectively determined by microsatellite analysis [33]. These included the DP, EK, LB, MB and BB field sites in southern Cameroon, the non-habituated Kalande (GM-KL) community in Gombe National Park, and a site in the Goualougo Triangle (GT), Republic of Congo [72]. At the remaining locations, the number of sampled chimpanzees remained unknown. These included the MF, MP, WE, MT, DG, BQ and CP field sites in Cameroon, the ME site in the Central African Republic, a site in the Lope National Park (LP) in Gabon, the BD, WL and WK field sites in the DRC, and a site in the Nyungwe Forest Reserve (NY) in Rwanda. Finally, samples were also obtained from the Ngogo community in Kibale National Park (KB). Although the Ngogo chimpanzees are habituated [52], the particular individuals sampled for this study were not identified.

### Other primates

Fecal samples were also obtained from a wild-living gorilla (LP5) and mandrill (LP47) in the Lope National Park as well as a wild-living bonobo (LM183) in the DRC. The species origin of these samples was confirmed by mtDNA analysis (Table S2). In addition, SFV *pol*-IN sequences were amplified from uncultured PBMC DNA from a wild-caught DeBrazza monkey (99CM-CNE1) previously reported to also harbor SIVdeb [73].

### Detection of SFVcpz specific antibodies in fecal extracts

Fecal samples were examined for the presence of SFVcpz specific antibodies using an enhanced chemiluminescent Western immunoblot assay modified for RNA*later* preserved specimens as described [33]. RNA*later* is a high salt solution (25 mM Sodium Citrate, 10 mM EDTA, 70 g ammonium sulfate/100 ml solution, pH 5.2) that preserves nucleic acids, but precipitates proteins, including immunoglobulin. To prepare extracts suitable for Western blot analysis, fecal/RNAlater mixtures (1.5 ml) were diluted with PBS-Tween-20 (8.5 ml), inactivated for 1 hr at 60°C, clarified by centrifugation (3500×*g* for 30 min) to remove solid debris, and then dialyzed against PBS overnight at 4°C to resuspend fecal immunoglobulin. Reconstituted extracts were subjected to immunoblot analysis using SFVcpz antigen containing strips.

For Western blot strip preparation, an infectious SFVcpz proviral clone (pMod-1) was transfected into BHK21 cells and the resulting virus expanded in Cf2Th cells [61],[74]. Briefly, Cf2Th cells (2×10^6^ cells per 150 mm dish) were inoculated using a multiplicity of infection of 0.1, harvested at 75–100% CPE, pelleted, and resuspended in PBS (30 ml). Virions were released from cells by repeated freezing and thawing (4 cycles), purified by ultracentrifugation through a 20% sucrose cushion (23,500×g, 2 hrs), analyzed for protein content using a protein assay kit (Pierce, Rockford, Ill.), denatured in Reducing Sample Buffer (Piece, Rockford, Ill.), boiled, and run on a 7.5% Criterion Ready Gel (BioRad, Hercules, Calif.). Proteins were transferred to polyvinylidene difluoride (PVDF) membranes (BioRad, Hercules, CA), which were cut into strips (4 mm width), incubated with blocking buffer (5% nonfat dry milk, 3% fetal bovine sera, 0.5% Tween-20 in PBS), and then reacted overnight at 4°C with fecal extracts as described [33]. Protein-bound antibody was detected with goat-anti-human IgG-HRP (Southern Biotech, Birmingham, AL) and Western blots were developed using an enhanced chemiluminescence (ECL) detection system (GE Healthcare Bio-Sciences, Piscataway, NJ).

### Amplification of SFVcpz sequences from fecal nucleic acids

Fecal RNA was extracted using the RNA*queous*-Midi kit (Applied Biosystems/Ambion, Austin, TX) and subjected to reverse transcriptase polymerase chain reaction (RT-PCR) amplification using different sets of SFVcpz specific primers. In each case, cDNA was synthesized using the outer reverse primer (R1), followed by nested PCR using forward (F1/F2) and reverse (R1/R2) primers. Fecal DNA was extracted using the QIAamp DNA Stool Mini Kit (Qiagen, Valencia, CA) and subjected to nested PCR. Previously described sets of nested primers were used to amplify subgenomic *pol*-IN (425 bp), *gag* (616 bp) and LTR (260 bp) regions [8], [10]–[13],[18]. In addition, nested *pol*-RT primers were designed to amplify a 717 bp reverse transcriptase (RT) fragment that extended the *pol*-IN fragment by 580 bp to the 5′ end (F1: 5′-AGCAGGATATGTAAGATATTATAATGA -3′; R1: 5′-TCTCATATTTGGCCACCAATAAAGG -3′ F2: 5′- TTTCATTATGATAAAACCTTACCAGAA -3′; R2: 5′- TCCGGTGTGAGCCAAATTGTGGGCTTG -3′). For a subset of samples, we also used forward *pol*-RT primers in combination with reverse *pol*-IN primers to amplify a 1,005 bp L-*pol* fragment. The positions of these primers in the SFV genome are shown in Figure 3. PCR conditions included 60 cycles of denaturation (94°C, 20 s), annealing (50°C, 30 s), and elongation (68°C, 1 min) for the first round. Second round conditions included 55 cycles of denaturation (94°C, 20 s), annealing (52°C, 30 s), and elongation (68°C, 1 min). Amplified products were gel purified (Qiagen) and sequenced directly without interim cloning. Population sequences were analyzed using Sequencher version 4.6 (Gene Codes Corporation, Ann Arbor, MI) and chromatograms were carefully examined for positions of base mixtures.

### Single genome amplification of SFVcpz sequences

For a subset of samples suspected to harbor SFVcpz recombinants or mixtures of distinct viral strains, the complexity of the SFVcpz viral population within individual hosts was independently analyzed by single genome amplification (SGA). Fecal RNA was extracted from additional aliquots and cDNA synthesized as described above. cDNA was endpoint diluted in 96-well plates such that fewer than 29 reactions yielded an amplification product. According to a Poisson distribution, the cDNA dilution that yields PCR products in no more than 30% of wells contains one amplifiable cDNA template per positive PCR more than 80% of the time. PCR conditions and primers were as described above. All amplicons were sequenced directly, and sequences with ambiguous positions excluded from further analysis.

### Sensitivity and specificity of SFVcpz antibody and nucleic acid detection

The sensitivities of SFVcpz antibody and viral nucleic acid detection were determined for captive (YK) as well as wild-living chimpanzees (TA, DP, EK, BB, MB, LB, GT, GM, MH) of known SFVcpz infection status. Captive chimpanzees were diagnosed as SFVcpz infected by demonstrating virus specific antibodies in their blood (Tables 1 and 2). Wild-living chimpanzees were identified as SFVcpz infected by demonstrating virus specific antibodies or viral RNA in at least one fecal sample (Table 2). For each site, sensitivities were calculated as the fraction of positive tests per total number of samples tested, with confidence limits determined given the assumption of binomial sampling. For these calculations, it was assumed that successive test results from the same individual were not correlated. The specificity of fecal antibody detection was calculated using test results from SFVcpz antibody negative human volunteers (Table 1) and determined to be 1.00 (0.87–1.00). The specificities of vRNA and vDNA detection in fecal samples were also 1.00, since all amplification products were sequence confirmed.

### SFVcpz prevalence estimations

For sites where the number of sampled chimpanzees was known (TA, DP, EK, BB, MB, LB, GT, GM-KL, GM-MT, GM-KK, MH), SFVcpz prevalence rates were estimated based on the proportion of infected individuals. For each chimpanzee, the probability that it would be detected as being infected, if it was truly infected, was calculated taking into consideration the sensitivities of the types of assays performed as well as the numbers of specimens analyzed. Since the sensitivities of antibody and viral RNA detection varied extensively between captive and wild chimpanzees as well as different collection sites (Table 2), test sensitivities were averaged across all field sites. These “field sensitivities” were then used to calculate SFVcpz prevalence rates, with 95% confidence limits determined based on binomial sampling.

For field sites where the number of sampled individuals was not known (MF, MP, WE, MT, DG, CP, BQ, LP, ME, BD, WL, WK, KB, NY), prevalence rates were estimated based on the number of fecal samples collected and tested. Based on results from previous field studies [33], it was assumed that a fraction (17%) of all fecal samples was partially degraded and that any given chimpanzee was sampled on average 1.72 times. Using these corrections, the proportion of SFVcpz infected chimpanzees was estimated for each field site, again taking into account the “field sensitivities” of the different tests as well as the numbers of specimens analyzed. In addition, the number of unique mtDNA haplotypes served as an indicator of the minimum number of chimpanzees analyzed. From these determinations, prevalence rates and their confidence limits were calculated.

### Species and subspecies determinations

The species and subspecies origin of all chimpanzee fecal samples used in this study was determined by mitochondrial (mt) DNA analysis (Table S1). A 498-bp region of the mtDNA genome (D loop) was amplified using primers L15997 (5′-CACCATTAGCACCCAAAGCT-3′) and H16498 (5′-CCTGAAGTAGGAACCAGATG-3′), and all amplification products were sequenced directly. The resulting sequences were aligned and identical sequences grouped into mtDNA haplotypes. A subset of these haplotypes has been reported previously [33]. The remainder were classified based on their phylogenetic relatedness to subspecies specific mtDNA reference sequences. Haplotypes and corresponding GenBank accession numbers are listed in Table S1.

### Phylogenetic analysis

Nucleotide sequences of SFVcpz *gag*, *pol*-RT *and pol*-IN fragments were aligned using Se-Al (A. Rambaut, distributed by the author at http://tree.bio.ed.ac.uk/software/seal/). Several previously characterized SFVcpz strains were included as reference sequences (GenBank accession numbers: SVFpvrc679, AY195683 and AY195708; SFVprvc1138, AY195682; SFVpvlcpz2, AY195686; SFVpvlcpz4, AY195687; CpzCam32, AY639133; CpzCam19, AY639141; CpzCam21, AY639122; SFVpsc925, AY195676 and AY195702; HFV, NC001795; SFVptr1040, AY195673 and AY195699; SFVptr1436, AY195700; SFVptrb1, AY195681 and AY195707; SFVcase6, AY195712; SFVpsc5126, AY195701 and AY195675; SFVpvra101, AY195678; SFVpvra055, AY195677; SFVpts-No, AJ627552; SFVpts-Ni, AJ627553; SFVcase14, AY195719; SFVcase13, AY195718; SFVpvra182, AY195706; SVFcase10, AY195716; SFVcase8, AY195714; SFVpvra136, AY195705; SFVcase9, AY195715; SFVcase12, AY195717; SFVcase7, AY195713; SFVpvrc941, AY195685 and AY195709; SFVcpz, NC001364; CpzCam44, AY639136; CpzCam30, AY639128; CpzCam15, AY639138; CpzCam35, AY639130; PanGabNto, AY639123; PanGabNte, AY639124; PanGabBel, AY639126; Ppan1935, AJ627551; SFV-6, X83296; SFV-7, X83297). Very few insertions or deletions were required to align the data, and the resulting gaps were treated as unknown characters. All the alignments are available from the authors upon request.

We initially used the Bayesian Markov chain Monte Carlo (BMCMC) method implemented in MrBayes v3.1 [75] to infer phylogenies for the mtDNA and SFV data. However, for some data sets, most notably the *pol*-IN alignment, we observed that the MCMC samples were dominated by trees that exhibited clearly spurious branching patterns, with long branches leading to distantly-related clades often breaking up the monophyly of closely related groups of sequences (not shown). We therefore employed the ‘relaxed molecular clock’ BMCMC method implemented in BEAST [76], so-called because it relaxes the assumption of a constant rate of evolution across the tree, allowing different lineages to evolve at different rates. Although our interest was not in estimating divergence dates, Drummond and colleagues found that using a model that falls between the extremes of assuming either a strict molecular clock or no molecular clock appeared to improve both the accuracy and precision of topology inference across a wide range of taxa [77]. Our results provide further support for this conclusion, since the artifacts described above for the MrBayes analyses were not observed in the BEAST results.

All the BEAST runs were performed under an uncorrelated lognormal relaxed molecular clock model with a constant population size coalescent tree prior, using a general time-reversible nucleotide substitution model with heterogeneity among sites modeled with a gamma distribution. For each mtDNA and SFVcpz data set, simultaneous sampling times were assumed since the small intervals between sampling dates are expected to be negligible given the long time span of evolution represented not only by the mtDNA but also the SFV data sets [13].

For each analysis, two independent runs of 5 to 20 million steps were performed. Examination of the MCMC samples with Tracer v1.4 (A. Rambaut & A. J. Drummond, http://beast.bio.ed.ac.uk) indicated convergence and adequate mixing of the Markov chains, with estimated sample sizes in the 100s or 1000s. After inspection with Tracer, we discarded an appropriate number of steps from each run as burn-in, and combined the resulting MCMC tree samples for subsequent estimation of posteriors. We summarized the MCMC samples using the maximum clade credibility (MCC) tree (including branch lengths) found using TreeAnnotator v1.4.7 [76], with posterior probabilities indicated (as percentages) for nodes with P>0.90. All trees were saved with branch lengths measured in substitutions per site rather than time.

### Recombination and co-infection analyses

In order to investigate the possibility of recombination in SFVcpz, and to map any putative recombination breakpoints, we conducted a recombination detection analysis using GENECONV [78]. GENECONV performs a series of comparisons between all pairs of sequences in an alignment and asks whether certain fragments are unusually alike (available from the author at http://www.math.wustl.edu/sawyer/geneconv/). For example, if two sequences are nearly identical over one stretch of sequence, but are highly divergent across the remainder, the similar fragment might be detected by GENECONV as a putative mosaic region. If, after statistically correcting for multiple comparisons, that fragment still appears to be unexpectedly similar, it will be flagged as a globally significant fragment by GENECONV. A simple follow-up analysis with phylogenetic trees inferred from the different regions detected by GENECONV can then confirm whether certain sequences contain regions with conflicting evolutionary histories (i.e. supporting significantly discordant topologies).

GENECONV results on a concatenated alignment of strains for which *gag*, *pol*-IN, and *pol*-RT sequences were available indicated several globally significant fragments; however, because many of the inferred breakpoints were at the *gag*-*pol* concatenation junction, we investigated the possibility that the putative “recombinants” detected with these data set actually represented co-infected samples in which different variants had been amplified for the distinct regions comprising the concatenated data set. Because this appeared to be the case, we restricted subsequent recombination analyses to individually amplified gene regions.

### Nucleotide sequence accession numbers

All newly obtained SFVcpz and mtDNA D-loop sequences have been submitted to GenBank, and accession numbers are listed in Tables S1 and S2, respectively.

## Supporting Information

Figure S1 Subspecies origin of chimpanzee fecal samples. Mitochondrial DNA sequences (498 bp D loop fragment) from SFVcpz positive chimpanzee fecal specimens were grouped into unique haplotypes (Table S1) and then compared to subspecies specific reference sequences by phylogenetic analysis. Sequences were analyzed using the Bayesian Markov chain Monte Carlo (BMCMC) method implemented in BEAST. The maximum clade credibility (MCC) topology is shown, with posterior probabilities (expressed as percentages) indicated on nodes depicted either as asterisks (100%) or filled circles (90%–99%). Haplotypes are color coded according to their subspecies origin (a box denotes a *P. t. troglodytes* haplotype identified in the range of *P. t. vellerosus*). The scale bar represents 0.002 substitutions per site.
(0.86 MB EPS)

Table S1 Mitochondrial DNA analysis of primate fecal samples.
(0.29 MB DOC)

Table S2 GenBank accession numbers of newly obtained SFV sequences.
(2.50 MB DOC)
