# Supplementary material for: Molecular Ecology and Natural History of Simian Foamy Virus Infection in Wild-Living Chimpanzees
Source: PLoS Pathog. 2008 Jul 4;4(7):e1000097. doi: 10.1371/journal.ppat.1000097 (PMC2435277; doi:10.1371/journal.ppat.1000097)
Supplement: Table S1 — Mitochondrial DNA analysis of primate fecal samples. (0.29 MB DOC) [file ppat.1000097.s002.doc]

| **Table S1.** Mitochondrial DNA Analysis of Primate Fecal Samples | | |
| --- | --- | --- |
| **Haplotype^a^** | **Fecal sample with identical haplotype^b^** | **GenBank accession number** |
| ME2514 | **ME2514, ME2518** | EU527407 |
| ME2515 | **ME2515** | EU527406 |
| ME2516 | **ME2516, ME2517** | EU527402 |
| ME2519 | **ME2519, ME2523,** ME2533 | EU527405 |
| ME2520 | **ME2520** | EU527408 |
| ME2521 | **ME2521**, ME2525, **ME2527, ME2534, ME2535,** **ME2554, ME2556** | EU527401 |
| ME2524 | ME2524 | EU527417 |
| ME2526 | ME2526 | EU527418 |
| ME2532 | ME2522, **ME2532** | EU527404 |
| ME2536 | **ME2536** | EU527403 |
| WL98 | WL98 | EU527444 |
| WL99 | WL99 | EU527454 |
| WL100 | WL100, WL101 | EU527447 |
| WL102 | WL102 | EU527452 |
| WL103 | WL103 | EU527450 |
| WL104 | WL104 | EU527451 |
| WL105 | WL105 | EU527448 |
| WL107 | WL107, WL115, WL124 | EU527453 |
| WL108 | WL108 | EU527445 |
| WL109 | WL109, WL118 | EU527446 |
| WL111 | WL106, WL111, WL112, WL113, WL125 | EU527455 |
| WL114 | WL114, WL116 | EU527449 |
| WL117 | **WL117** | EU527388 |
| WK4 | WK3, **WK4,** WK5, WK10 | EU527385 |
| WK7 | WK6, **WK7,** WK14, KB28, KB75 | EU527386 |
| WK8 | **WK8,** WK9 | EU527387 |
| WK12 | WK12, WK17 | EU527456 |
| BD2 | BD1, **BD2,** BD3, BD4 | EU527391 |
| BD5 | **BD5** | EU527392 |
| BD6 | BD6 | EU527427 |
| BD8 | BD8 | EU527428 |
| BD10 | **BD10** | EU527393 |
| BD12 | **BD12** | EU527394 |
| BD13 | BD11, **BD13, BD15,** BD17 | EU527395 |
| BD14 | **BD14** | EU527396 |
| BD16 | BD16 | EU527429 |
| TA1 | TA1 | EU527424 |
| TA3 | **TA3,** TA16, **TA20** | DQ370354 |
| TA6 | **TA6** | EU527378 |
| TA8 | **TA8** | EU527379 |
| TA9 | TA9 | EU527440 |
| TA10 | TA10 | EU527441 |
| TA11 | TA11 | EU527426 |
| TA17 | **TA17** | DQ370355 |
| TA19 | TA19 | EU527442 |
| TA21 | TA7, **TA21** | EU527380 |
| TA22 | TA22 | EU527425 |
| TA24 | **TA24** | EU527381 |
| TA25 | TA25 | EU527443 |
| LP1 | LP1, LP16 | EU527432 |
| LP7 | LP7 | EU527433 |
| LP9 | LP9 | EU527434 |
| LP14 | **LP14,** LP15, LP17 | EU527397 |
| LP20 | **LP20** | EU527398 |
| LP22 | LP22 | EU527435 |
| LP24 | LP24 | EU527436 |
| LP27 | LP27 | EU527437 |
| LP29 | **LP29** | EU527399 |
| LP48 | **LP48** | EU527400 |
| KB27 | KB27, KB68, KB69 | EU527463 |
| KB29 | KB29, KB33, KB65 | EU527458 |
| KB32 | KB32 | EU527459 |
| KB35 | **KB35,** KB39 | EU527382 |
| KB38 | KB38, KB43 | EU527464 |
| KB40 | KB26, KB30, KB40, KB41, KB64 | EU527462 |
| KB42 | KB42, KB66, NY399 | EU527457 |
| KB44 | KB36, KB37, **KB44,** KB59 | EU527383 |
| KB67 | KB34, KB67 | EU527460 |
| KB73 | KB73 | EU527461 |
| NY1 | NY1 | DQ370353 |
| NY2 | **NY2,** NY17, NY404 | DQ370351 |
| NY4 | NY4 | DQ370349 |
| NY6 | NY6, NY7, NY12, NY13, NY15, NY19 | DQ370350 |
| NY9 | NY9, NY408, NY411 | EU527438 |
| NY14 | NY10, NY11, **NY14,** NY20 | EU527389 |
| NY16 | NY8, **NY16, NY18,** NY410 | EU527390 |
| NY401 | NY401, NY402, NY406 | EU527465 |
| NY409 | NY409 | EU527466 |
| GM1 | GM111, GM144, GM233, GM241, GM251, GM327, **GM428** | DQ370324 |
| GM2 | GM226, **GM231,** GM238, GM245, GM247, GM230, GM248, GM270, GM282, GM317 | DQ370331 |
| GM3 | GM170, GM220, GM240, GM289, **GM694** | DQ370319 |
| GM4 | GM620 | DQ370320 |
| GM5 | GM122, GM333, GM352, **GM445,** GM673 | DQ370326 |
| GM6 | GM13, GM35, GM166, **GM167,** GM214, GM225, GM275, GM284, GM340, GM357, **GM338,** GM487, **GM498,** GM625 | DQ370327 |
| GM8 | GM108, GM119, GM154, GM173, **GM235,** GM249, GM250, GM706, **GM707, GM708** | DQ370322 |
| GM10 | GM242, **GM278,** GM298, GM299, GM314, GM316, GM374 | DQ370328 |
| GM11 | GM27, **GM82,** GM97, GM186, **GM188, GM199,** GM207, GM216, GM224, GM271, GM300, GM326, GM336, **GM666, GM667** | DQ370325 |
| GM13 | GM228, GM256, GM257, GM281, GM297, GM301, GM302 | DQ370323 |
| MH32 | MH32, MH35, MH43, MH51, MH56, MH57, MH62, MH63, MH70 | EU527468 |
| MH37 | MH37, MH42, MH58, MH60, MH66 | EU527467 |
| MH41 | MH29, MH30, **MH41**, MH59, MH67, MH68 | EU527384 |
| GT1 | **GT305,** GT308, GT313 | DQ370316 |
| GT2 | GT303, **GT307,** GT314, **GT319** | DQ370315 |
| GT3 | GT302, GT304, GT309, **GT310**, GT315, GT318 | DQ370318 |
| GT4 | **GT311,** GT312 | DQ370317 |
| GT301 | GT301 | EU527430 |
| GT316 | GT316 | EU527431 |
| MG159 | **SA161,** MG159 | DQ370308 |
| MG163 | **SA163** | DQ370310 |
| MF1269 | **MF1269** | EU527376 |
| MF1271 | MF1271, MF1280 | EU527419 |
| MF1274 | **MF1274** | EU527372 |
| MF1278 | **MF1278,** MF1290, **MF1293** | EU527377 |
| MF1279 | **MF1279, MF1281** | EU527370 |
| MF1289 | MF1289 | EU527420 |
| MF1297 | **MF1297** | EU527371 |
| MF1300 | MF1300 | EU527421 |
| MF1302 | MF1302 | EU527422 |
| MP1309 | MP1309 | EU527423 |
| MP1310 | **MP1310, MP1315** | EU527375 |
| MP1314 | **MP1314** | EU527373 |
| MP1345 | **MP1345** | EU527374 |
| UB446 | **UB446** | EU527411 |
| KS310 | **KS310** | EU527413 |
| EP479 | **EP479** | EU527412 |
| EP486 | **EP486** | EU527416 |
| BA432 | **BA432** | EU527410 |
| BF1167 | **BF1167** | EU527414 |
| WA466 | **WA466** | EU527409 |
| WA543 | **WA543** | EU527415 |
| CM1 | BQ28, BQ33, BQ50, **MT150**, MT330, BQ497, BQ499, DP03, DP17, **DP18,** DP19, DP22, DP23, DP26, DP27, DP67, DP69, DP80, DP82, DP130, DP134, DP163, DP206, DP218, DP220 | DQ367534 |
| CM2 | BQ29 | DQ367535 |
| CM3 | BQ30 | DQ367536 |
| CM4 | BQ32, BQ44, MT122, MT123, MT125, MT153, MT156, **MT157**, BQ193, **BB241,** MT335, MT371, MT373, MT436, MT437, EK504, EK508, EK509, DP01, **DP05,** DP103, DP104, DP137, DP151, **DP159,** DP162 | DQ367537 |
| CM5 | BQ38, BQ389, BQ391, BQ392, MB135, LB188, EK503, EK517, DP98, DG533, **DG541** | DQ367538 |
| CM6 | BQ39, BQ42, BQ52, BQ55, **BQ474,** BQ481, BQ484, BQ487 | DQ367539 |
| CM7 | **BQ40,** BQ41, MT326, **MT337,** MT339, **DG407,** **CP384,** EK513, **EK522** | DQ367540 |
| CM8 | BQ43, BQ51, BQ83, BQ84, BQ387, BQ388, BQ393, MT334, DP14, DP83, DP89, DP90, DP91, DP92, DP94, **DP99,** DP100, DP101, **DP110**, **DP140**, DP142, DP219 | DQ367541 |
| CM9 | BQ45, BQ47, MT121, MB140, MT141, LB310, MT348, MT357, MT365, MT385, **BQ390**, BQ396, BQ397, BQ398, BQ400, BQ401, BQ402, BQ403, BQ473, BQ475, BQ482, BQ489, BQ494, BQ495, BQ496, BQ498, BQ500, **DG524** | DQ367542 |
| CM10 | BQ46, BQ194, BQ195, BQ490 | DQ367543 |
| CM11 | BQ48, **BQ57**, BQ394, **BQ476**, BQ483, BQ488, BQ488a, BQ491 | DQ367544 |
| CM12 | BQ49, MB97, DP70 | DQ367545 |
| CM13 | BQ54, **MB23**, MB24, **LB307**, LB308, BQ399, DP71, DP72, DP74, **DP75**, **DG546** | DQ367546 |
| CM14 | BQ56, **BQ59**, BQ471, BQ477, BQ478, BQ479, BQ480, BQ485 | DQ367547 |
| CM15 | MT51, MT52, MT126 | DQ367548 |
| CM16 | MT53, MT54, MT120, MT124, MT154, MT155, MT343, MT344, MT345, MT346, MT347, MT349, MT350, MT351, MT353, MT354, MT355, MT356, MT358, MT360, MT361, MT362, MT363, MT366, MT386 | DQ367549 |
| CM17 | **MB66** | DQ367550 |
| CM18 | BB72, BB75, BB79, **BB104** | DQ367551 |
| CM19 | BB73, **BB74**, BB87, **BB94**, BB102, **BB230**, **DG526**, DG542, DG543 | DQ367552 |
| CM20 | BB76, **BB239**, **EK501** | DQ367553 |
| CM21 | **BB77** | DQ367554 |
| CM22 | BB78, BB101, BB103, MT342 | DQ367555 |
| CM23 | **BQ81**, MT127, LB204, **LB205**, LB208, MT329, **MT331**, MT332, MT333, MT336, MT340, DP62, **DP65**, **DG531, DG547, DG548, DG549, DG562** | DQ367556 |
| CM24 | **BQ82**, EK521, DP63, DP64, DP68, **DP127**, DP128, DP131, DP132, | DQ367557 |
| CM25 | **BQ85**, BQ86, GT306, GT317, GT320 | DQ367558 |
| CM26 | BB88, BB95 | DQ367559 |
| CM27 | BB89, BB237, BB240 | DQ367560 |
| CM28 | BB99, BB100, **LB174**, LB175 | DQ367561 |
| CM29 | BB93, BB229, **BB234**, BB238 | DQ367562 |
| CM30 | **BB106** | DQ367563 |
| CM31 | MT114, MT115, MT116, MT117, MT148 | DQ367564 |
| CM32 | MT119, MT151, EK516 | DQ367565 |
| CM33 | MB138, MB139, MB189, **MB191**, MB192, MB250, LB311, LB312, LB313, **MB318**, MB319, MB320 | DQ367566 |
| CM34 | MT142, MT143, MT145, MT147, MT149, LB176, MB324 | DQ367567 |
| CM35 | **MT144**, DP129, DP133 | DQ367568 |
| CM36 | MT146 | DQ367569 |
| CM37 | LB186 | DQ367570 |
| CM38^c^ | MB190, **LB309^c^** | DQ367571 |
| CM39 | **BB235** | DQ367572 |
| CM40 | BB236 | DQ367573 |
| CM41 | MB245 | DQ367574 |
| CM42 | **MB246**, MB247, MB248, **EK511** | DQ367575 |
| CM43 | **MB315**, MB316 | DQ367576 |
| CM44 | MB317 | DQ367577 |
| CM45 | MB323 | DQ367578 |
| CM46 | MT325, **MT338, DG525, DG527, DG530, DG532, DG534, DG535** | DQ367579 |
| CM47 | **MT327** | DQ367580 |
| CM48 | MT341 | DQ367581 |
| CM49 | MT352, MT359, MT364 | DQ367582 |
| CM50 | MT379 | DQ367583 |
| CM51 | **CP380**, CP381, CP382 | DQ367584 |
| CM52 | **CP383** | DQ367585 |
| CM53 | BQ395, BQ404, BQ492, **BQ493** | DQ367586 |
| CM54 | **DG405**, DP78, DP79, DP88, **DP112**, DP113, DP114, DP115, DP116 | DQ367587 |
| CM55 | **DG406** | DQ367588 |
| CM56 | WE438, WE439, **WE440, WE441, WE442,** WE443, WE444, WE445, WE446, WE447, **WE448, WE449**, WE450, **WE451**, WE454, WE456, WE457, WE458, WE459, WE460, **WE461, WE462** | DQ367532 |
| CM57 | WE452, WE453 | DQ367533 |
| CM58 | WE455, **WE464** | DQ367589 |
| CM59 | **CP466** | DQ367590 |
| CM60 | **CP467**, EK502, **EK505, EK506**, EK507 | DQ367591 |
| CM61 | CP468, **EK510** | DQ367592 |
| CM62 | CP469 | DQ367593 |
| CM63 | **CP470** | DQ367594 |
| CM64 | BQ472, DG523, DG529, DG536, **DG537**, DG538 | DQ367595 |
| CM65 | **EK512** | DQ367596 |
| CM66 | EK518 | DQ367597 |
| CM67 | EK519 | DQ367598 |
| CM68 | **DG528, DG539** | DQ367599 |
| CM69 | **DG540** | DQ367600 |
| CM70 | **DP04** | DQ367601 |
| CM71 | DP06, DP13, DP102 | DQ367602 |
| CM72 | DP07, DP08, DP12, **DP16** | DQ367603 |
| CM73 | DP09, DP10, DP11, DP15, DP20, DP24, DP25, DP108, **DP109,** DP126 | DQ367604 |
| CM74 | DP66, DP154, DP156 | DQ367605 |
| CM75 | DP77, DP95, DP97 | DQ367606 |
| CM76 | **DP81,** DP85, **DP124** | DQ367607 |
| CM77 | **DP93**, DP96 | DQ367608 |
| CM78 | DP105, DP106, DP107, DP160 | DQ367609 |
| CM79 | DP136, **DP141**, DP143, DP144, DP145, DP146, DP148, DP149, DP150 | DQ367610 |
| CM80 | DP152, DP153, DP164, DP165 | DQ367611 |
| CM81 | DP155, **DP157**, DP158 | DQ367612 |
| CM82 | **LB7** | DQ367613 |
| YK2 | YK1, **YK2, YK3, YK5,** YK6, YK11, **YK13, YK14, YK15,** **YK20,** **YK32, YK34,** YK36, YK37, **YK39, YK41** | DQ370365 |
| YK4 | **YK4,** **YK12,** **YK8, YK9, YK10,** **YK17,** **YK29** | DQ370363 |
| YK16 | YK16 | DQ370358 |
| YK18 | **YK18,** YK19, YK21 | DQ370359 |
| YK22 | **YK22, YK24, YK25, YK28** | DQ370360 |
| YK23 | **YK23** | DQ370362 |
| YK26 | **YK26, YK27** | DQ370357 |
| YK30 | **YK30, YK31** | DQ370364 |
| YK33 | **YK33, YK35** | DQ370361 |
| YK38 | YK38 | EU527439 |
| YK40 | YK40 | DQ370356 |
| LP5^d^ | **LP5** | EU527469 |
| LP47^e^ | **LP47 (COII)** | EU527471 |
| LM183^f^ | **LM183** | EU527470 |

^a^All primate fecal samples were subjected to mtDNA analysis to confirm their species and subspecies origin, and to exclude specimen degradation. The resulting sequences (498 bp D loop fragment) were grouped into unique mtDNA haplotypes and submitted to GenBank. Samples are coded according to their field site of origin (as shown in Figure 1). YK denotes samples from captive chimpanzees housed at the Yerkes Regional Primate Research Center. Haplotypes CM1 - CM82 have been reported previously [33]. CM58 denotes a *P. t. troglodytes* haplotype identified in the range of *P. t. vellerosus* apes.

^b^SFVcpz antibody and/or nucleic acid positive samples are highlighted in red, with samples containing viral RNA sequences boldfaced (a phylogenetic tree of mtDNA sequences from the latter is shown in Figure S1).

^c^LB309 harbored an SFV strain from a *Cercopithecus* monkey species (Figure 10).

^d^LP5 was derived from a wild-living gorilla (*Gorilla gorilla*).

^e^LP47 was derived from a wild-living mandrill (*Mandrillus sphinx*); for this sample, cytochrome oxidase subunit II mtDNA sequences were determined.

^f^LM183 was derived from a wild-living bonobo (*Pan paniscus*).
