# Supplementary material for: Molecular Ecology and Natural History of Simian Foamy Virus Infection in Wild-Living Chimpanzees
Source: PLoS Pathog. 2008 Jul 4;4(7):e1000097. doi: 10.1371/journal.ppat.1000097 (PMC2435277; doi:10.1371/journal.ppat.1000097)
Supplement: Table S2 — GenBank accession numbers of newly obtained SFV sequences. (2.50 MB DOC) [file ppat.1000097.s003.doc]

Sequences are labeled by genomic regions (as depicted in Figure 3), with identical (=) and single genome amplified (SGA) sequences indicated. Sequences labeled with A and B were derived from different RNA extracts but from the same sample. Samples are coded according to their field site of origin (YK denotes captive chimpanzees from the Yerkes Regional Primate Research Center).

| **Table S2.** GenBank Accession Numbers of Newly Obtained SFV Sequences | | | | | | | | | |
| --- | --- | --- | --- | --- | --- | --- | --- | --- | --- |
| **SFVcpz**  ***pol-*IN**  **(425bp)** | **GenBank accession number** | **SFVcpz**  ***pol-*RT**  **(717bp)** | **GenBank accession number** | **SFVcpz**  **L*pol***  **(1,005bp)** | **GenBank accession number** | **SFVcpz**  ***gag***  **(616bp)** | **GenBank Accession Number** | **SFVcpz**  ***LTR***  **(260bp)** | **GenBank accession number** |
| YK2IN | EU527508 |  |  |  |  | YK2GAG | EU527602 | YK2LTR | EU527672 |
| YK3IN | EU527507 |  |  |  |  | YK3GAG | EU527603 |  |  |
| YK4IN | =YK3IN |  |  |  |  |  |  |  |  |
| YK8IN | =YK3IN |  |  |  |  |  |  |  |  |
| YK9IN | =YK3IN |  |  |  |  |  |  |  |  |
| YK10IN | =YK3IN |  |  |  |  |  |  |  |  |
| YK12IN | =YK3IN |  |  |  |  |  |  |  |  |
| YK13IN | =YK3IN |  |  |  |  |  |  |  |  |
| YK14IN | =YK3IN |  |  |  |  |  |  |  |  |
| YK39IN | =YK3IN |  |  |  |  |  |  |  |  |
| YK41IN | =YK3IN |  |  |  |  |  |  |  |  |
| YK5IN | EU527504 |  |  |  |  |  |  |  |  |
| YK17IN | =YK5IN |  |  |  |  |  |  |  |  |
| YK15IN | EU527515 |  |  |  |  |  |  |  |  |
| YK33IN | =YK15IN |  |  |  |  |  |  |  |  |
| YK34IN | =YK15IN |  |  |  |  |  |  |  |  |
| YK35IN | =YK15IN |  |  |  |  |  |  |  |  |
| YK18IN | EU527517 |  |  |  |  |  |  |  |  |
| YK20IN | =YK18IN |  |  |  |  |  |  |  |  |
| YK22IN | EU527513 |  |  |  |  |  |  |  |  |
| YK24IN | =YK22IN |  |  |  |  |  |  |  |  |
| YK25IN | =YK22IN |  |  |  |  |  |  |  |  |
| YK28IN | =YK22IN |  |  |  |  |  |  |  |  |
| YK23IN | EU527518 |  |  |  |  |  |  |  |  |
| YK26IN | EU527525 |  |  |  |  |  |  |  |  |
| YK27IN | =YK26IN |  |  |  |  |  |  |  |  |
| YK29IN | EU527526 |  |  |  |  |  |  |  |  |
| YK30IN | EU527520 |  |  |  |  |  |  |  |  |
| YK31IN | =YK30IN |  |  |  |  |  |  |  |  |
| YK32IN | EU527519 |  |  |  |  |  |  |  |  |
| TA3IN | EU527506 |  |  |  |  |  |  |  |  |
| TA6IN | EU527503 |  |  |  |  |  |  |  |  |
| TA8IN | EU527510 | TA8RT | EU527691 |  |  |  |  |  |  |
| TA21IN | =TA8IN |  |  |  |  |  |  |  |  |
| TA17IN | EU527505 |  |  |  |  | TA17GAG | EU527609 |  |  |
| TA20IN | =YK2IN |  |  |  |  |  |  |  |  |
| TA24IN | EU527509 |  |  |  |  | TA24GAG | EU527610 |  |  |
| WE440IN | EU527493 |  |  |  |  |  |  | WE440LTR | EU527661 |
| WE441IN | =WE440IN |  |  |  |  |  |  | WE441LTR | =WE440LTR |
| WE461IN | =WE440IN |  |  |  |  |  |  |  |  |
|  |  |  |  |  |  |  |  | WE448LTR | =WE440LTR |
| WE451IN | EU527492 | WE451RT | EU527673 |  |  | WE451GAG | EU527598 |  |  |
| WE442IN | =WE451IN |  |  |  |  |  |  | WE442LTR | EU527660 |
| WE449IN | =WE451IN |  |  |  |  |  |  | WE449LTR | EU527662 |
| WE462IN | =WE451IN |  |  |  |  | WE462GAG | EU527658 |  |  |
| WE464IN | EU527539 | WE464RT | EU527674 |  |  | WE464GAG | EU527599 |  |  |
| SA161IN | EU527487 |  |  |  |  |  |  |  |  |
| SA163IN | EU527528 |  |  |  |  |  |  | SA163LTR | EU527664 |
| MF1269IN | =MF1269Lpol | MF1269RT | =MF1269Lpol | MF1269Lpol | EU527474 | MF1269GAG | EU527646 |  |  |
| MF1274IN | =MF1274Lpol | MF1274RT | =MF1274Lpol | MF1274Lpol | EU527478 | MF1274GAG | EU527653 |  |  |
| MF1279IN | =MF1279Lpol | MF1279A-RT | =MF1279Lpol | MF1279Lpol | EU527480 | MF1279GAG | EU527652 |  |  |
| MF1279IN-SGA-A6 | EU582052 |  |  |  |  |  |  |  |  |
| MF1279IN-SGA-A10 | EU582053 |  |  |  |  |  |  |  |  |
| MF1279IN-SGA-A1 | =A10 |  |  |  |  |  |  |  |  |
| MF1279IN-SGA-A2 | =A10 |  |  |  |  |  |  |  |  |
| MF1279IN-SGA-A3 | =A10 |  |  |  |  |  |  |  |  |
| MF1279IN-SGA-A4 | =A10 |  |  |  |  |  |  |  |  |
| MF1279IN-SGA-A5 | =A10 |  |  |  |  |  |  |  |  |
| MF1279IN-SGA-A9 | =A10 |  |  |  |  |  |  |  |  |
| MF1279IN-SGA-B1 | =A10 |  |  |  |  |  |  |  |  |
| MF1279IN-SGA-B4 | =A10 |  |  |  |  |  |  |  |  |
| MF1279IN-SGA-B5 | =A10 |  |  |  |  |  |  |  |  |
| MF1279IN-SGA-B6 | =A10 |  |  |  |  |  |  |  |  |
| MF1279IN-SGA-B8 | =A10 |  |  |  |  |  |  |  |  |
| MF1279IN-SGA-C2 | =A10 |  |  |  |  |  |  |  |  |
| MF1279IN-SGA-C7-1 | =A10 |  |  |  |  |  |  |  |  |
| MF1279IN-SGA-C10 | =A10 |  |  |  |  |  |  |  |  |
| MF1279IN-SGA-D6 | =A10 |  |  |  |  |  |  |  |  |
| MF1279IN-SGA-D10 | =A10 |  |  |  |  |  |  |  |  |
| MF1279IN-SGA-B9 | EU582051 |  |  |  |  |  |  |  |  |
| MF1279IN-SGA-C5 | EU582047 |  |  |  |  |  |  |  |  |
| MF1279IN-SGA-C6 | EU582048 |  |  |  |  |  |  |  |  |
| MF1279IN-SGA-C7-2 | EU582049 |  |  |  |  |  |  |  |  |
| MF1279IN-SGA-D3 | EU582050 |  |  |  |  |  |  |  |  |
| MF1279IN-SGA-A8 | =D3 |  |  |  |  |  |  |  |  |
| MF1279IN-SGA-B10 | =D3 |  |  |  |  |  |  |  |  |
|  |  | MF1279B-RT | EU527675 |  |  |  |  |  |  |
| MF1281IN | =MF1279Lpol | MF1281RT | =MF1279Lpol | MF1281Lpol | =MF1279Lpol | MF1281GAG | =MF1279GAG |  |  |
| MF1278IN | =MF1278Lpol | MF1278RT | =MF1278Lpol | MF1278Lpol | EU527479 |  |  |  |  |
| MF1293IN | =MF1278Lpol | MF1293RT | =MF1278Lpol |  |  |  |  |  |  |
| MF1297IN | =MF1278Lpol |  |  | MF1297Lpol | =MF1278Lpol | MF1297GAG | EU527654 |  |  |
| MP1315IN | EU527585 |  |  |  |  | MP1315GAG | EU527655 |  |  |
| MP1310IN | =MP1315IN |  |  |  |  |  |  |  |  |
| MP1314IN | EU527584 |  |  |  |  |  |  |  |  |
| MP1345IN | =MP1345Lpol | MP1345RT | =MP1345Lpol | MP1345Lpol | EU527476 | MP1345GAG | EU527656 |  |  |
| DP4IN | =DG534Lpol |  |  |  |  | DP4GAG | EU527614 |  |  |
| DP5IN | =EK511IN |  |  |  |  | DP5GAG | =EK522GAG |  |  |
| DP16IN | =EK511IN |  |  |  |  |  |  |  |  |
| DP93IN | =EK511IN |  |  |  |  |  |  |  |  |
| DP127IN | =EK511IN |  |  |  |  | DP127GAG | =EK522GAG |  |  |
| DP18IN | EU527549 |  |  |  |  | DP18GAG | EU527638 |  |  |
| DP65IN | EU527550 | DP65RT | EU527686 |  |  |  |  |  |  |
| DP75IN | =MB318IN |  |  |  |  |  |  |  |  |
| DP81IN | EU527551 |  |  |  |  |  |  |  |  |
| DP99IN | EU527572 |  |  |  |  | DP99GAG | EU527617 |  |  |
| DP110IN | =DP99IN |  |  |  |  |  |  | DP110LTR | EU527665 |
| DP109IN | EU527484 |  |  |  |  |  |  |  |  |
| DP112IN | EU527554 |  |  |  |  | DP112GAG | =MB318GAG |  |  |
| DP124IN | EU527555 |  |  |  |  |  |  |  |  |
| DP140IN | EU527556 |  |  |  |  |  |  |  |  |
| DP141IN | EU527557 |  |  |  |  | DP141GAG | EU527631 |  |  |
| DP157A-IN | EU527481 | DP157A-RT | EU527677 |  |  | DP157GAG | EU527639 |  |  |
| DP157B-IN | EU527482 | DP157B-RT | EU527676 |  |  |  |  |  |  |
| DP157IN-SGA-A12 | EU582043 |  |  |  |  |  |  |  |  |
| DP157IN-SGA-A32 | =A12 |  |  |  |  |  |  |  |  |
| DP157IN-SGA-A71 | =A12 |  |  |  |  |  |  |  |  |
| DP157IN-SGA-A72 | =A12 |  |  |  |  |  |  |  |  |
| DP157IN-SGA-C71 | =A12 |  |  |  |  |  |  |  |  |
| DP157IN-SGA-B12 | EU582045 |  |  |  |  |  |  |  |  |
| DP157IN-SGA-B62 | EU582044 |  |  |  |  |  |  |  |  |
| DP157IN-SGA-B51 | =B62 |  |  |  |  |  |  |  |  |
| DP157IN-SGA-B71 | EU582042 |  |  |  |  |  |  |  |  |
| DP157IN-SGA-C32 | EU582046 |  |  |  |  |  |  |  |  |
| DP157IN-SGA-A42 | =C32 |  |  |  |  |  |  |  |  |
| DP157IN-SGA-B112 | =C32 |  |  |  |  |  |  |  |  |
| DP157IN-SGA-C42 | EU582041 |  |  |  |  |  |  |  |  |
| DP157IN-SGA-D11 | EU582040 |  |  |  |  |  |  |  |  |
| DP157IN-SGA-D12 | EU582039 |  |  |  |  |  |  |  |  |
|  |  | DP158RT | =DP157A-RT |  |  |  |  |  |  |
| DP159IN | EU527558 |  |  |  |  | DP159GAG | =EK522GAG |  |  |
| BQ40IN | EU527546 |  |  |  |  | BQ40GAG | EU527621 |  |  |
| BQ57IN | =MB318IN |  |  |  |  |  |  | BQ57LTR | EU527666 |
| BQ59IN | =MB318IN |  |  |  |  | BQ59GAG | EU527622 |  |  |
| BQ474IN | =MB318IN |  |  |  |  | BQ474GAG | =BQ59GAG |  |  |
| BQ476IN | =MB318IN |  |  |  |  |  |  |  |  |
| BQ493IN | =MB318IN |  |  |  |  | BQ493GAG | =BQ59GAG |  |  |
| BQ81IN | =DP81IN |  |  |  |  | BQ81GAG | EU527623 |  |  |
| BQ82IN | =EK511IN |  |  |  |  | BQ82GAG | =EK522GAG |  |  |
| BQ85IN | EU527547 |  |  |  |  |  |  |  |  |
| BQ390IN | =EK510IN |  |  |  |  |  |  |  |  |
| DG405IN | EU527523 |  |  |  |  |  |  |  |  |
| DG406IN | EU527521 |  |  |  |  |  |  |  |  |
| DG407IN | EU527569 |  |  |  |  | DG407GAG | EU527640 |  |  |
| DG524IN | EU527553 | DG524RT | =DP65RT |  |  |  |  |  |  |
| DG534IN | =DG534Lpol | DG534A-RT | =DG534Lpol | DG534Lpol | EU527473 | DG534GAG | EU527629 |  |  |
|  |  | DG534B-RT | EU527678 |  |  |  |  |  |  |
| DG525IN | =DG534Lpol |  |  |  |  | DG525GAG | =DG534GAG |  |  |
| DG527IN | =DG534Lpol | DG527RT | EU527679 |  |  | DG527GAG | =DG534GAG |  |  |
| DG530IN | =DG534Lpol |  |  |  |  | DG530GAG | =DG534GAG |  |  |
| DG532IN | =DG534Lpol |  |  |  |  | DG532GAG | =DG534GAG |  |  |
| DG535IN | =DG534Lpol | DG535RT | =DG527RT |  |  | DG535GAG | =DG534GAG |  |  |
| DG540IN | =DG534Lpol |  |  |  |  |  |  |  |  |
| DG526IN | EU527568 |  |  |  |  | DG526GAG | EU527630 |  |  |
| DG541IN | =DG526IN |  |  |  |  |  |  |  |  |
| DG528IN | EU527567 |  |  |  |  | DG528GAG | EU527643 |  |  |
| DG539IN | =DG528IN |  |  |  |  |  |  |  |  |
| DG531IN | EU527566 |  |  |  |  |  |  |  |  |
| DG562IN | =DG531IN |  |  |  |  | DG562GAG | EU527632 |  |  |
| DG537IN | EU527565 |  |  |  |  |  |  |  |  |
| DG549IN | EU527564 | DG549RT | EU527687 |  |  | DG549GAG | EU527633 |  |  |
| DG547IN | =DG549IN |  |  |  |  |  |  |  |  |
| DG548IN | =DG549IN |  |  |  |  | DG548GAG | =DG549GAG |  |  |
| DG546IN | =DP109IN |  |  |  |  |  |  |  |  |
| CP380IN | =DP99IN |  |  |  |  | CP380GAG | EU527624 |  |  |
| CP383IN | =EK511IN |  |  |  |  | CP383GAG | EU527625 |  |  |
| CP384IN | EU527571 |  |  |  |  | CP384GAG | EU527626 |  |  |
| CP466IN | EU527516 |  |  |  |  | CP466GAG | EU527605 |  |  |
| CP467IN | EU527570 |  |  |  |  | CP467GAG | EU527627 |  |  |
| CP470IN | =CP470Lpol | CP470RT | =CP470Lpol | CP470Lpol | EU527472 | CP470GAG | EU527628 |  |  |
| EK511IN | EU527552 | EK511RT | EU527682 |  |  | EK511GAG | EU527636 |  |  |
| EK501IN | =EK511IN |  |  |  |  |  |  |  |  |
| EK505IN | EU527563 | EK505RT | =CP470RT |  |  | EK505GAG | EU527642 |  |  |
| EK506IN | =EK505IN | EK506RT | EU527684 |  |  | EK506GAG | =EK505GAG |  |  |
| EK510IN | EU527576 | EK510RT | EU527693 |  |  |  |  |  |  |
| EK512IN | EU527562 |  |  |  |  | EK512GAG | EU527612 |  |  |
| EK522IN | EU527561 | EK522RT | EU527683 |  |  | EK522GAG | EU527637 |  |  |
| BB94IN | EU527578 |  |  |  |  | BB94GAG | EU527615 |  |  |
| BB74IN | =BB94IN |  |  |  |  |  |  |  |  |
| BB230IN | =BB94IN |  |  |  |  | BB230GAG | =BB94GAG |  |  |
| BB77IN | =EK510IN |  |  |  |  | BB77GAG | EU527641 |  |  |
| BB104IN | =EK511IN |  |  |  |  | BB104GAG | EU527616 |  |  |
| BB106IN | =EK511IN |  |  |  |  |  |  |  |  |
| BB234IN | EU527575 |  |  |  |  | BB234GAG | EU527618 |  |  |
| BB235IN | EU527574 |  |  |  |  | BB235GAG | EU527619 |  |  |
| BB239IN | =MB66IN |  |  |  |  | BB239GAG | EU527620 |  |  |
| BB241IN | EU527573 |  |  |  |  |  |  |  |  |
| MB23IN | EU527483 | MB23RT | EU527680 |  |  |  |  |  |  |
| MB66IN | EU527514 | MB66RT | EU527695 |  |  |  |  |  |  |
| MB246IN | =MB66IN |  |  |  |  | MB246GAG | EU527659 |  |  |
| MB191IN | EU527577 | MB191RT | EU527688 |  |  | MB191GAG | EU527634 |  |  |
| MB315IN | =DP99IN |  |  |  |  | MB315GAG | EU527613 |  |  |
| MB318IN | EU527548 | MB318RT | EU527681 |  |  | MB318GAG | EU527635 |  |  |
| LB7IN | =MB66IN | LB7RT | EU527690 |  |  |  |  |  |  |
| LB174IN | =DP99IN |  |  |  |  | LB174GAG | EU527611 |  |  |
| LB205IN | EU527560 |  |  |  |  |  |  |  |  |
| LB307IN | EU527559 |  |  |  |  | LB307GAG | =MB246GAG |  |  |
| MT144IN | EU527488 |  |  |  |  | MT144GAG | EU527607 | MT144LTR | EU527663 |
| MT150IN | EU527494 |  |  |  |  |  |  |  |  |
| MT157IN | EU527511 |  |  |  |  |  |  | MT157LTR | EU527668 |
| MT327IN | EU527495 |  |  |  |  |  |  |  |  |
| MT331IN | EU527512 |  |  |  |  |  |  | MT331LTR | EU527667 |
| MT337IN | EU527529 |  |  |  |  |  |  |  |  |
| MT338IN | EU527524 |  |  |  |  |  |  |  |  |
| ME2514IN | EU527582 | ME2514RT | EU527698 |  |  | ME2514GAG | EU527657 |  |  |
| ME2515IN | =ME2514IN |  |  |  |  |  |  |  |  |
| ME2516IN | =ME2514IN |  |  |  |  | ME2516GAG | =ME2517GAG |  |  |
| ME2518IN | =ME2514IN |  |  |  |  | ME2518GAG | EU527651 |  |  |
| ME2517IN | EU527583 | ME2517RT | EU527697 |  |  | ME2517GAG | EU527644 |  |  |
| ME2520IN | =ME2520Lpol |  |  | ME2520Lpol | EU527475 | ME2520GAG | EU527648 |  |  |
| ME2532IN | =ME2520Lpol |  |  |  |  | ME2532GAG | EU527647 |  |  |
| ME2521IN | EU527579 | ME2521RT | EU527696 |  |  | ME2521GAG | EU527645 |  |  |
| ME2527IN | =ME2521IN |  |  |  |  |  |  |  |  |
| ME2554IN | =ME2521IN |  |  |  |  | ME2554GAG | =ME2521GAG |  |  |
| ME2556IN | =ME2521IN | ME2556RT | EU527699 |  |  | ME2556GAG | =ME2521GAG |  |  |
| ME2523IN | =ME2523Lpol |  |  | ME2523Lpol | EU527477 | ME2523GAG | EU527650 |  |  |
| ME2519IN | =ME2523Lpol | ME2519RT | =ME2523Lpol |  |  | ME2519GAG | =ME2523GAG |  |  |
| ME2534IN | EU527581 |  |  |  |  | ME2534GAG | =ME2521GAG |  |  |
| ME2535IN | =ME2534IN |  |  |  |  | ME2535GAG | =ME2521GAG |  |  |
| ME2536IN | EU527580 |  |  |  |  | ME2536GAG | EU527649 |  |  |
| GT305IN | EU527489 |  |  |  |  |  |  |  |  |
| GT307IN | =ME2523Lpol |  |  |  |  |  |  |  |  |
| GT319IN | =ME2523Lpol |  |  |  |  |  |  |  |  |
| GT310IN | EU527490 |  |  |  |  |  |  |  |  |
| GT311IN | EU527491 |  |  |  |  |  |  |  |  |
| LP14IN | EU527586 |  |  |  |  |  |  |  |  |
| LP20IN | EU527589 |  |  |  |  |  |  |  |  |
| LP29IN | EU527587 | LP29RT | EU527694 |  |  | LP29GAG | EU527606 |  |  |
| LP48IN | EU527588 |  |  |  |  |  |  |  |  |
| BD2IN | EU527531 |  |  |  |  |  |  |  |  |
| BD5IN | EU527530 |  |  |  |  |  |  |  |  |
| BD12IN | =BD5IN |  |  |  |  |  |  |  |  |
| BD10IN | EU527532 |  |  |  |  | BD10GAG | EU527604 |  |  |
| BD15IN | =BD10IN |  |  |  |  |  |  |  |  |
| BD13IN | EU527533 |  |  |  |  |  |  |  |  |
| BD14IN | =BD13IN |  |  |  |  |  |  |  |  |
| WL117IN | EU527527 |  |  |  |  |  |  |  |  |
| WK4IN | EU527501 |  |  |  |  |  |  |  |  |
| WK7IN | EU527500 |  |  |  |  |  |  |  |  |
| WK8IN | EU527499 |  |  |  |  |  |  |  |  |
| BA432IN | EU527542 |  |  |  |  |  |  |  |  |
| BF1167IN | EU527592 |  |  |  |  |  |  |  |  |
| EP479IN | EU527545 |  |  |  |  |  |  |  |  |
| EP486IN | EU527590 |  |  |  |  |  |  |  |  |
| KS310IN | EU527541 |  |  |  |  |  |  |  |  |
| UB446IN | EU527543 |  |  |  |  |  |  |  |  |
| WA466IN | EU527544 |  |  |  |  |  |  |  |  |
| WA543IN | EU527591 |  |  |  |  |  |  |  |  |
| GM199IN | EU527485 | GM199RT | EU527685 |  |  | GM199GAG | EU527601 |  |  |
| GM82IN | =GM199IN |  |  |  |  | GM82GAG | =GM199GAG |  |  |
| GM167IN | =GM199IN |  |  |  |  |  |  |  |  |
| GM188IN | =GM199IN |  |  |  |  |  |  |  |  |
| GM231IN | =GM199IN |  |  |  |  |  |  | GM231LTR | EU527669 |
| GM445IN | =GM199IN |  |  |  |  |  |  |  |  |
| GM666IN | =GM199IN |  |  |  |  | GM666GAG | =GM199GAG |  |  |
| GM667IN | =GM199IN |  |  |  |  | GM667GAG | =GM199GAG |  |  |
| GM235IN | EU527486 |  |  |  |  |  |  |  |  |
| GM338IN | EU527502 |  |  |  |  |  |  |  |  |
| GM428IN | EU527497 |  |  |  |  |  |  |  |  |
| GM498IN | EU527496 |  |  |  |  |  |  |  |  |
| GM694IN | EU527538 |  |  |  |  |  |  |  |  |
| GM708IN | EU527498 | GM708RT | EU527692 |  |  | GM708GAG | EU527600 |  |  |
| GM278IN | =GM708IN |  |  |  |  |  |  | GM278LTR | EU527670 |
| GM707IN | =GM708IN |  |  |  |  |  |  |  |  |
| KB35IN | EU527537 |  |  |  |  | KB35GAG | EU527608 |  |  |
| KB44IN | EU527540 |  |  |  |  |  |  |  |  |
| MH41IN | EU527522 |  |  |  |  |  |  |  |  |
| NY2IN | EU527535 |  |  |  |  |  |  |  |  |
| NY18IN | =NY2IN |  |  |  |  |  |  |  |  |
| NY14IN | EU527534 | NY14RT | EU527689 |  |  |  |  |  |  |
| NY16IN | EU527536 |  |  |  |  |  |  |  |  |
| LB309IN^a^ | EU527596 |  |  |  |  |  |  |  |  |
| LM183IN^b^ | EU527595 | LM183RT | EU527700 |  |  |  |  |  |  |
| LP5IN^c^ | EU527593 |  |  |  |  |  |  |  |  |
| LP47IN^d^ | EU527594 |  |  |  |  |  |  |  |  |
| CNE1IN^e^ | EU527597 |  |  |  |  |  |  | CNE1LTR | EU527671 |

^a^LB309 *pol*-IN sequences represent an SFV strain from a *Cercopithecus* monkey species (Figure 10).

^b^LM183 *pol*-IN and *pol*-RT sequences were amplified from fecal RNA of a wild-living bonobo (*Pan paniscus*).

^c^LP5 *pol*-IN sequences were amplified from fecal RNA of a wild-living gorilla (*Gorilla gorilla*).

^d^LP47 *pol*-IN sequences were amplified from fecal RNA of a wild-living mandrill (*Mandrillus sphinx*).

^e^CNE1 *pol*-IN sequences were amplified from peripheral blood mononuclear cell DNA of a wild-living DeBrazza’s monkey (*Cercopithecus neglectus*) [73].
